# The pro-inflammatory effects of combined exposure to diesel exhaust particles and mineral particles in human bronchial epithelial cells

**DOI:** 10.1186/s12989-022-00455-0

**Published:** 2022-02-21

**Authors:** Vegard Sæter Grytting, Prem Chand, Marit Låg, Johan Øvrevik, Magne Refsnes

**Affiliations:** Section of Air Quality and Noise, Department of Environmental Health, Norwegian Institute of Public Health, PO box 4404, 0403 Nydalen, Oslo, Norway

**Keywords:** Air pollution, Combustion particles, Stone particles, Quartz, Minerals, Inflammation, Particulate matter, Pulmonary, Epithelium, Cytokines

## Abstract

**Background:**

People are exposed to ambient particulate matter (PM) from multiple sources simultaneously in both environmental and occupational settings. However, combinatory effects of particles from different sources have received little attention in experimental studies. In the present study, the pro-inflammatory effects of combined exposure to diesel exhaust particles (DEP) and mineral particles, two common PM constituents, were explored in human lung epithelial cells.

**Methods:**

Particle-induced secretion of pro-inflammatory cytokines (CXCL8 and IL-1β) and changes in expression of genes related to inflammation (CXCL8, IL-1α, IL-1β and COX-2), redox responses (HO-1) and xenobiotic metabolism (CYP1A1 and CYP1B1) were assessed in human bronchial epithelial cells (HBEC3-KT) after combined exposure to different samples of DEP and mineral particles. Combined exposure was also conducted using lipophilic organic extracts of DEP to assess the contribution of soluble organic chemicals. Moreover, the role of the aryl hydrocarbon receptor (AhR) pathway was assessed using an AhR-specific inhibitor (CH223191).

**Results:**

Combined exposure to DEP and mineral particles induced increases in pro-inflammatory cytokines and expression of genes related to inflammation and redox responses in HBEC3-KT cells that were greater than either particle sample alone. Moreover, robust increases in the expression of CYP1A1 and CYP1B1 were observed. The effects were most pronounced after combined exposure to α-quartz and DEP from an older fossil diesel, but enhanced responses were also observed using DEP generated from a modern biodiesel blend and several stone particle samples of mixed mineral composition. Moreover, the effect of combined exposure on cytokine secretion could also be induced by lipophilic organic extracts of DEP. Pre-incubation with an AhR-specific inhibitor reduced the particle-induced cytokine responses, suggesting that the effects were at least partially dependent on AhR.

**Conclusions:**

Exposure to DEP and mineral particles in combination induces enhanced pro-inflammatory responses in human bronchial epithelial cells compared with exposure to the individual particle samples. The effects are partly mediated through an AhR-dependent pathway and lipophilic organic chemicals in DEP appear to play a central role. These possible combinatory effects between different sources and components of PM warrant further attention and should also be considered when assessing measures to reduce PM-induced health effects.

**Supplementary Information:**

The online version contains supplementary material available at 10.1186/s12989-022-00455-0.

## Introduction

During the last decades, firm links have been established between exposure to ambient particulate matter (PM) and mortality and morbidity due to cardiovascular and respiratory diseases [1–6]. Moreover, inhalation of various types of PM remains a common cause of occupational lung diseases worldwide [7]. Ambient PM consists of a complex mixture of particles derived from multiple sources, which vary spatially and temporally [8–10]. Likewise, the workplace atmosphere may contain particles from multiple sources, depending on the occupation and the tasks performed [11–13]. Although exposure to multiple sources of particles may occur concomitantly, experimental studies mainly focus on individual PM components or sources of PM, or on mixed ambient PM samples, and rarely assess combinations of particles from different sources in a systematic way. While studies suggest that combined exposure to PM and other environmental factors may induce increased effects compared with the individual compounds [14–18], the combination of different types of PM has received little attention and warrants further studies to fully understand the impact of complex particle mixtures on human health.

Diesel exhaust particles (DEP) are generated through combustion of diesel fuel by vehicles or diesel-powered equipment and exposure occurs in both environmental and occupational settings. Particularly high levels of exposure have been detected in underground mining and tunnelling operations [19]. DEP consists of a carbon nanoparticle core with a complex mixture of adhered metals and organic chemicals, and the composition varies depending on the fuel mixture, drive cycle, operating conditions and combustion engine technology [20–22]. Controlled exposure studies show that exposure to diesel exhaust can cause pulmonary and systemic inflammation, endothelial dysfunction, and increased airway resistance and hyperreactivity in human volunteers [23–26]. These findings are supported by experimental studies showing that DEP exposure induces pro-inflammatory responses in vivo and in vitro [20, 27, 28]. Diesel exhaust is also considered a human carcinogen [29]. The effects of DEP are often attributed to soluble organic PM constituents, such as polycyclic aromatic hydrocarbons (PAH), which may induce inflammatory responses in part through aryl hydrocarbon receptor (AhR)-dependent mechanisms [30, 31].

Respirable mineral particles also represent a potential health hazard in several occupational settings and exposure may occur in conjunction with DEP due to the use of diesel-powered equipment, for instance in mining and tunnelling operations [11, 12, 19, 32–34]. Moreover, minerals may be major components of ambient PM and can originate from both natural sources and human activity [35–37]. Traffic represents an important source of mineral-rich particles in cities due to abrasion of the road surface and resuspension of road dust [38, 39]. This is especially problematic in Nordic countries due to the widespread use of studded tyres during winter, which increases road-surface abrasion [36, 38, 40]. While the mineralogy of road dust and ambient PM is not commonly assessed, some studies have reported the presence of several different types of minerals, including quartz, feldspars, mica and various clay minerals [41–43]. However, the contribution of the mineral fraction to PM-induced diseases is not fully known. Inhalation of crystalline silica, usually in the form of α-quartz, is a well-characterized health hazard and is associated with pulmonary diseases such as silicosis, chronic obstructive pulmonary disease and lung cancer [44–46]. While other minerals have received less attention, previous studies from our group suggest that a variety of mineral particle samples can induce pro-inflammatory responses in vitro and in vivo, and that the potency of the particles differs between samples of different mineral composition [47–50]. Moreover, recent studies show that several mineral particle samples can induce cytotoxicity and cytokine release of the same magnitude as quartz in immune cells and bronchial epithelial cells in vitro [48]. Furthermore, studies using road wear simulators suggest that mineral-rich particles from asphalt composed of different stone materials also vary in their ability to induce cytokine release in macrophages [51, 52], suggesting that the presence of different stone materials in asphalt may possibly confer different toxic properties to the resulting PM.

As both DEP and mineral particles are common constituents of air pollution in environmental and occupational settings their relative potency and potential combinatory effects in terms of inducing human health effects warrant further attention. In the present study, the pro-inflammatory effects of combined exposure to DEP and mineral particles were assessed in human airway epithelial cells to shed light on possible combinatory effects between these types of particles and whether these effects exceed the effects of the individual particle samples.

## Results

### Combined exposure to α-quartz and diesel exhaust particles induces secretion of pro-inflammatory cytokines in human bronchial epithelial cells

Three DEP samples of different origin and composition were selected for the present study. DEP_B0_ was generated by an old diesel engine running on gas oil [53], while DEP_B7_ and DEP_B20_ were generated by a more modern diesel engine running on 1st generation biodiesel fuel blends containing 7% and 20% fatty acid methyl ester (FAME), respectively, and represent the DEP more commonly encountered today [21]. The endotoxin content of the DEP_B0_, DEP_B7_ and DEP_B20_ samples were 1.2, 8.6 and 7.2 EU/mg, respectively. Preliminary experiments showed that exposure to DEP_B0_, DEP_B7_ and DEP_B20_ caused significant increases in CXC-motif chemokine ligand 8 (CXCL8) and interleukin (IL)-1β secretion (Additional file 1: Figure S1) in hTERT and CDK4 immortalized human bronchial epithelial cells (HBEC3-KT). Overall, DEP_B0_ induced the highest maximum responses followed by DEP_B7_, while DEP_B20_ was the least potent. The responses tended to increase up to 50 μg/mL, the only exception being IL-1β secretion induced by DEP_B0_ which peaked at 200 μg/mL. At higher concentrations the responses decreased almost to control levels, which coincided with changes in cell metabolic activity, suggesting that the viability of the cells was affected at these concentrations (Additional file 1: Figure S1). Based on these results, the concentrations of 25 and 50 µg/mL DEP (2.6 and 5.3 μg/cm^2^) were chosen for the combination experiments. The concentrations of 25, 50 and 100 μg/mL α-quartz (2.6, 5.3 and 10.5 μg/cm^2^) were chosen based on previous experiments published elsewhere [48]. A cytokine-binding assay was also performed to assess whether non-specific binding of cytokines to DEP may have interfered with the measurements [54]. At 50 µg/mL, the DEP_B0_ sample bound higher amounts of both CXCL8 and IL-1β compared with the DEP_B7_ and DEP_B20_ samples, suggesting that the differences in cytokine responses between these samples may be somewhat underestimated using the current methodology (Additional file 2: Figure S2).

**Fig. 1:**
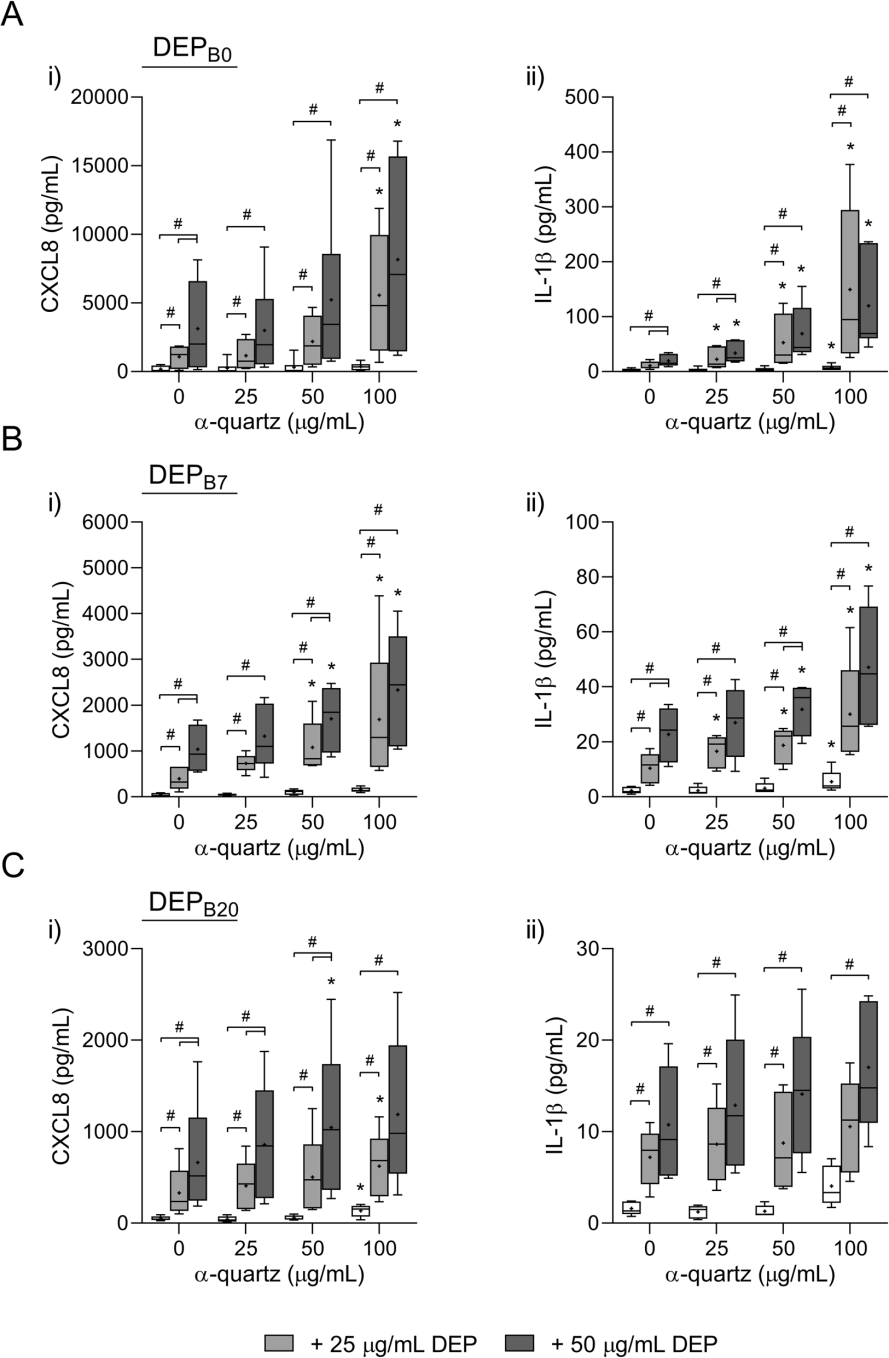
Cytokine responses after combined exposure to DEP and α-quartz. HBEC3-KT cells were exposed for 24 h to 25–50 μg/mL (2.6–5.3 μg/cm^2^) DEP_B0_ (**A**), DEP_B7_ (**B**) and DEP_B20_ (**C**), and 25–100 μg/mL (2.6–10.5 μg/cm^2^) α-quartz, alone or in combination. The release of CXCL8 (i) and IL-1β (ii) in the cell culture supernatants was measured using ELISA. Results are presented as boxplots of 6 independent experiments (Box: 25th–75th percentile, whiskers: minimum and maximum values, line: median, +: mean). Statistically significant differences were determined using two-way repeated measures ANOVA followed by Dunnett’s and Tukey post-tests. All values were log-transformed prior to statistical analysis to satisfy model assumptions. *Statistically significant difference from the respective control at 0 μg/mL α-quartz. #Statistically significant difference between exposure groups

Next, HBEC3-KT cells were exposed to increasing concentrations of α-quartz and DEP, both alone and in combination, to assess possible combinatory effects between the particle samples. While neither α-quartz nor DEP_B0_ alone decreased the viability of the HBEC3-KT cells at these concentrations, combined exposure caused a small decrease in viability that culminated at approximately 10% mean reduction in metabolic activity at the highest concentrations (Data not shown). However, no statistically significant decrease in cell viability was detected after exposure to DEP_B7_ or DEP_B20_ in combination with α-quartz (Data not shown). The combination of α-quartz and DEP_B0_ induced significantly higher cytokine responses than the response induced by α-quartz alone (Fig. 1a). Moreover, the combination of DEP_B0_ and increasing concentrations of α-quartz increased the particle-induced secretion of CXCL8 and IL-1β compared with exposure to DEP_B0_ alone, suggesting that α-quartz may enhance the effect of DEP_B0_ on cytokine release (Fig. 1a). Although the responses were lower in magnitude, combined exposure to DEP_B7_ and α-quartz also induced a concentration-dependent increase in CXCL8 and IL-1β secretion that was significantly higher than the effects of either particle sample alone (Fig. 1b). In comparison, DEP_B20_ induced lower levels of cytokine secretion in combination with α-quartz than both DEP_B0_ and DEP_B7_ (Fig. 1c). While the combination of α-quartz and DEP_B20_ induced a significantly higher response than α-quartz alone at all concentrations, only a small increase in CXCL8 and IL-1β secretion compared with DEP_B20_ alone was detected (Fig. 1c). As the DEP_B20_ sample induced lower levels of cytokines than DEP_B0_ and DEP_B7_, this sample was not included in further experiments.

### The combination of α-quartz and DEP induces the expression of genes related to inflammation and redox responses

To characterize the particle-induced responses further, the expression of a panel of genes related to inflammation and redox responses was assessed after 2, 6 and 12 h exposure to the DEP samples alone or in combination with α-quartz. CXCL8, IL-1β and IL-1α are pro-inflammatory cytokines that activate the inflammatory response in recipient cells and recruit circulating immune cells [55], while cyclooxygenase (COX)-2 is an enzyme involved in the generation of pro-inflammatory prostaglandins [56]. Heme oxygenase (HO)-1 was included as a marker for particle-induced redox responses [57]. Based on the results in Fig. 1, 50 and 100 µg/mL α-quartz and 25 µg/mL DEP_B0_ and DEP_B7_ were chosen for the gene expression analysis.

**Fig. 2:**
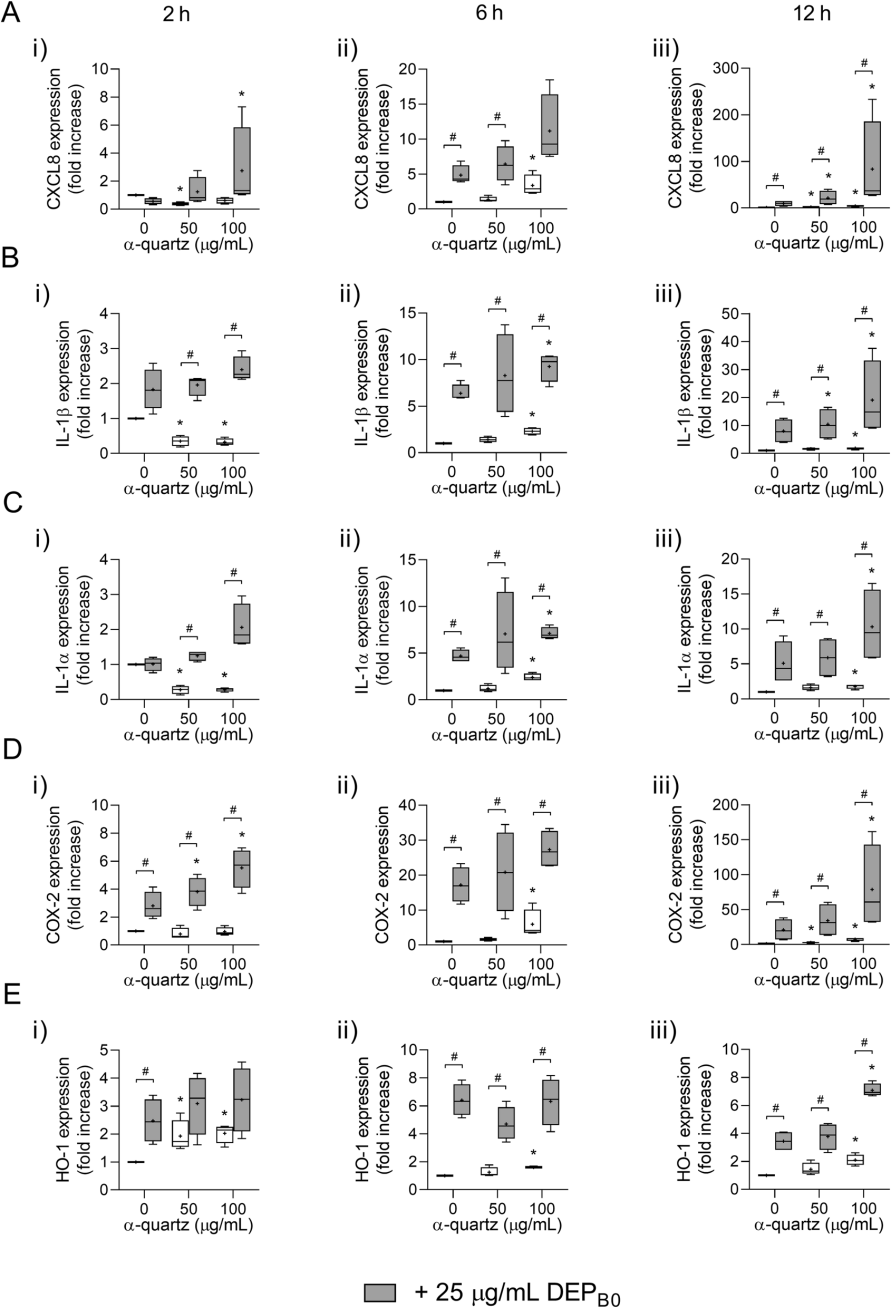
Expression of genes related to inflammation and redox responses after combined exposure to DEP_B0_ and α-quartz. HBEC3-KT cells were exposed to 25 μg/mL (2.6 μg/cm^2^) DEP_B0_ and 50–100 μg/mL (5.3–10.5 μg/cm^2^) α-quartz for 2 h (i), 6 h (ii) and 12 h (iii), alone or in combination. The expression of CXCL8 (**A**), IL-1β (**B**), IL-1α (**C**), COX-2 (**D**) and HO-1 (**E**) was determined using qPCR. Results are expressed as fold change compared to unexposed cells and presented as boxplots of 4 independent experiments (Box: 25th-75th percentile, whiskers: minimum and maximum values, line: median, +: mean). Statistically significant differences were determined using two-way repeated measures ANOVA followed by Dunnett’s and Šídák's post-tests and were calculated from the normalized CT values. *Statistically significant difference from the respective control at 0 μg/mL α-quartz. #Statistically significant difference between exposure groups

Exposure to DEP_B0_ and α-quartz induced a time-dependent increase in CXCL8, IL-1β, IL-1α and COX-2 expression that was highest after 12 h exposure (Fig. 2a–d). As observed for cytokine release, expression was significantly higher after combined exposure to α-quartz and DEP_B0_ compared to the individual effects of the two particle samples (Fig. 2a–d). Interestingly, combined exposure to DEP_B0_ and α-quartz induced a significant increase in COX-2 expression after 2 h even though α-quartz did not induce any significant effect by itself (Fig. 2d). Similar trends were also observed for expression CXCL8, IL-1β and IL-1α, although the effects did not reach statistical significance in all cases (Fig. 2a–c). HO-1 expression was significantly increased after 2 h exposure to both α-quartz and DEP_B0_ and increased further after 6 h (Fig. 2e). After 12 h exposure, the combination of DEP_B0_ and α-quartz induced an additional increase in HO-1 expression that was significantly higher than after exposure to the individual particle samples (Fig. 2e).

**Fig. 3:**
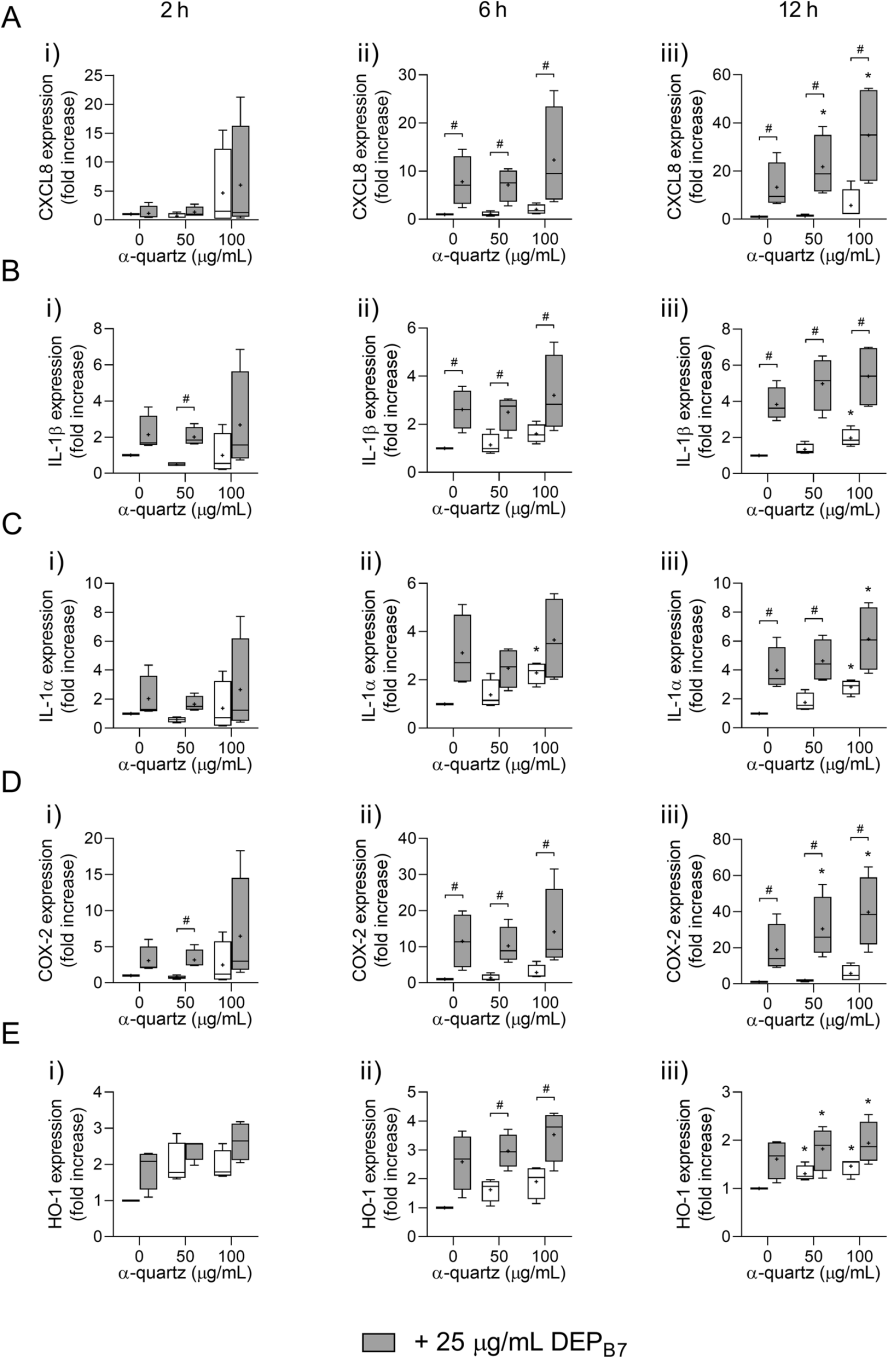
Expression of genes related to inflammation and redox responses after combined exposure to DEP_B7_ and α-quartz. HBEC3-KT cells were exposed to 25 μg/mL (2.6 μg/cm^2^) DEP_B7_ and 50–100 μg/mL (5.3–10.5 μg/cm^2^) α-quartz for 2 h (i), 6 h (ii) and 12 h (iii), alone or in combination. The expression of CXCL8 (**A**), IL-1β (**B**), IL-1α (**C**), COX-2 (**D**) and HO-1 (**E**) was determined using qPCR. Results are expressed as fold change compared to unexposed cells and presented as boxplots of 4 independent experiments (Box: 25th–75th percentile, whiskers: minimum and maximum values, line: median, +: mean). Statistically significant differences were determined using two-way repeated measures ANOVA followed by Dunnett’s and Šídák's post-tests and were calculated from the normalized CT values. *Statistically significant difference from the respective control at 0 μg/mL α-quartz. #Statistically significant difference between exposure groups

A time-dependent increase in gene expression was also observed after exposure to DEP_B7_ and α-quartz, which was highest after 12 h exposure for CXCL8, IL-1β, IL-1α and COX-2, and after 6 h exposure for HO-1 (Fig. 3). As observed for DEP_B0_, the combination of DEP_B7_ and α-quartz induced increased expression of CXCL8, IL-1β, IL-1α, COX-2 and HO-1 compared to the individual particle samples, although the effects were not as pronounced for all genes (Fig. 3). While a trend for increased expression was observed at earlier time points, the combinative effects of DEP_B7_ and α-quartz on CXCL8, IL-1α, COX-2 and HO-1 expression were not significantly different from DEP_B7_ alone until after 12 h exposure (Fig. 3). A similar trend was also observed for IL-1β, but the increase was not statistically significant (Fig. 3b). Moreover, the combinative effects of DEP_B7_ and α-quartz on IL-1α and HO-1 expression were not significantly different from α-quartz at the same concentration, although a trend for increased effects was observed (Fig. 3c, e).

### Exposure to DEP and α-quartz induces expression of xenobiotic metabolism enzymes CYP1A1 and CYP1B1

**Fig. 4:**
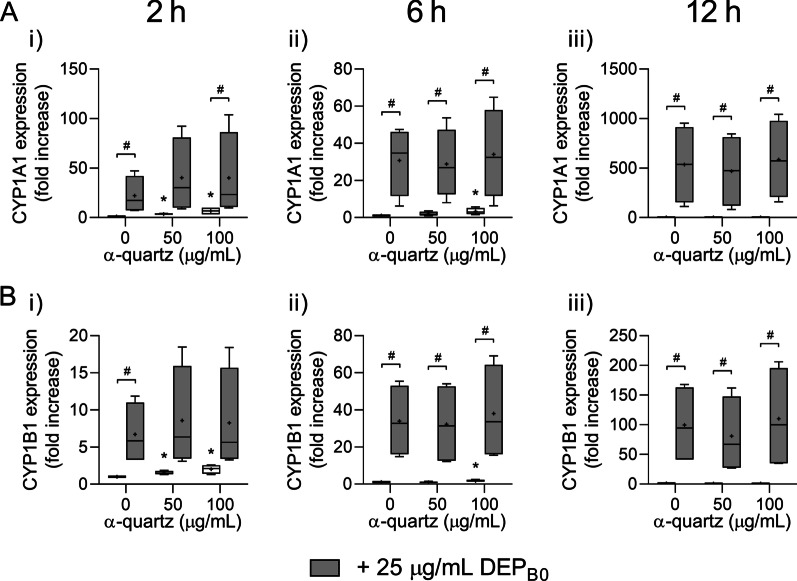
Expression of CYP1A1 and CYP1B1 after combined exposure to DEP_B0_ and α-quartz. HBEC3-KT cells were exposed to 25 μg/mL (2.6 μg/cm^2^) DEP_B0_ and 50–100 μg/mL (5.3–10.5 μg/cm^2^) α-quartz for 2 h (i), 6 h (ii) and 12 h (iii), alone or in combination. The expression of CYP1A1 (**A**) and CYP1B1 (**B**) was determined using qPCR. Results are expressed as fold change compared to unexposed cells and presented as boxplots of 4 independent experiments (Box: 25th–75th percentile, whiskers: minimum and maximum values, line: median, +: mean). Statistically significant differences were determined using two-way repeated measures ANOVA followed by Dunnett’s and Šídák's post-tests and were calculated from the normalized CT values. *Statistically significant difference from the respective control at 0 μg/mL α-quartz. #Statistically significant difference between exposure groups

Cytochrome p450 (CYP) 1A1 and CYP1B1 are enzymes involved in xenobiotic metabolism that may metabolically activate organic chemicals in DEP, such as PAHs, and are marker genes for canonical AhR-activation [58, 59]. Exposure to DEP_B0_ induced time-dependent increases in expression of CYP1A1 and CYP1B1, both alone and in combination with α-quartz (Fig. 4). While the responses were greatest after 12 h exposure, robust increases in the expression of both genes were detected already after 2 h. The effects on CYP expression were primarily induced by DEP_B0_, as evident by the small effect of α-quartz compared with DEP_B0_ and the similarity between the combination of particles and DEP_B0_ alone (Fig. 4). In contrast to the effects on genes related to inflammation and redox responses (Fig. 2), no significant difference in expression was detected between cells exposed to DEP_B0_ alone or in combination with α-quartz at any concentration or time point (Fig. 4).

**Fig. 5:**
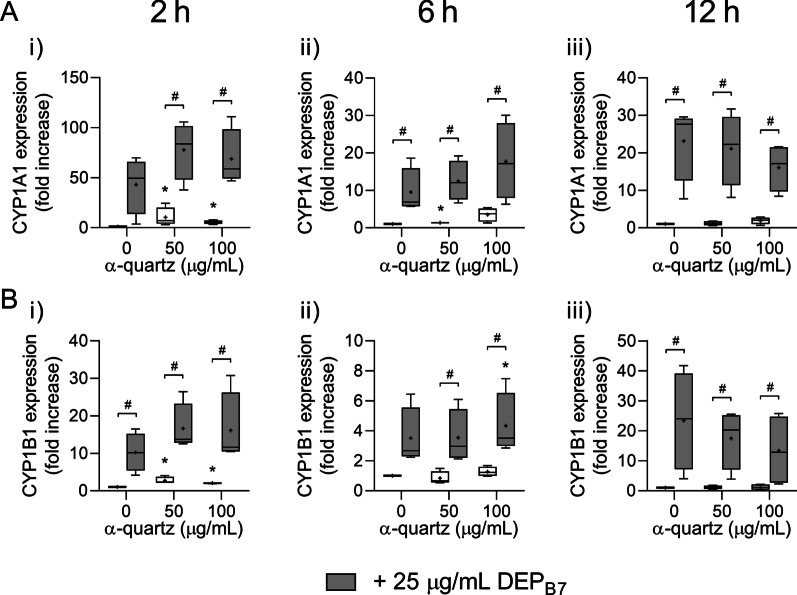
Expression of CYP1A1 and CYP1B1 after combined exposure to DEP_B7_ and α-quartz. HBEC3-KT cells were exposed to 25 μg/mL (2.6 μg/cm^2^) DEP_B7_ and 50–100 μg/mL (5.3–10.5 μg/cm^2^) α-quartz for 2 h (i), 6 h (ii) and 12 h (iii), alone or in combination. The expression of CYP1A1 (**A**) and CYP1B1 (**B**) was determined using qPCR. Results are expressed as fold change compared to unexposed cells and presented as boxplots of 4 independent experiments (Box: 25th–75th percentile, whiskers: minimum and maximum values, line: median, +: mean). Statistically significant differences were determined using two-way repeated measures ANOVA followed by Dunnett’s and Šídák's post-tests and were calculated based on normalized CT values. *Statistically significant difference from the respective control at 0 μg/mL α-quartz. #Statistically significant difference between exposure groups

Exposure to DEP_B7_ also induced an increase in the expression of both genes but appeared to follow a different time course than DEP_B0_. The CYP1A1 expression was highest after 2 h and decreased at 6–12 h (Fig. 5a). For CYP1B1 the effect was similar, but the expression was highest after 12 h exposure (Fig. 5b). As for DEP_B0_, no significant increase in CYP1A1 expression was detected after exposure to DEP_B7_ in combination with α-quartz, although a small concentration-dependent increase was observed after 2–6 h and a small decrease after 12 h (Fig. 5a). Similar results were observed for CYP1B1. However, the small increase observed at 6 h after combined exposure was significantly higher than the response induced by DEP_B7_ alone (Fig. 5b).

### The effects of DEP are partly dependent on the aryl hydrocarbon receptor pathway

**Fig. 6:**
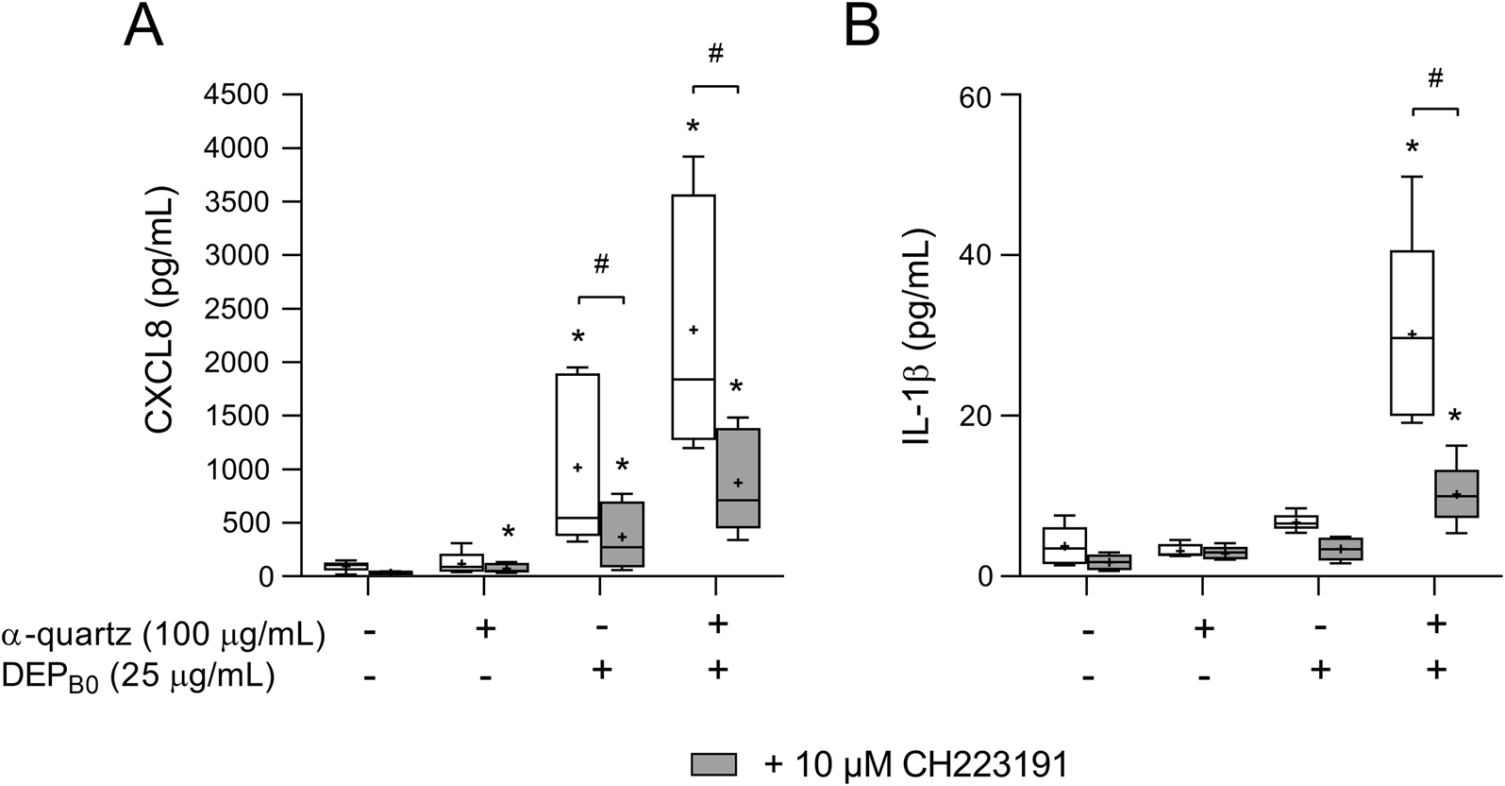
The effects of AhR-inhibition on the cytokine responses induced by combined exposure to DEP_B0_ and α-quartz. HBEC3-KT cells were exposed to the AhR-specific inhibitor CH223191 (10 μM) for 30 min prior to 24 h exposure to 25 μg/mL (2.6 μg/cm^2^) DEP_B0_ and 100 μg/mL (10.5 μg/cm^2^) α-quartz. The release of CXCL8 (**A**) and IL-1β (**B**) in the cell culture supernatants was measured using ELISA. Results are presented as boxplots of 5 independent experiments (Box: 25th–75th percentile, whiskers: minimum and maximum values, line: median, +: mean). Statistically significant differences were determined using two-way repeated measures ANOVA followed by Dunnett’s and Šídák's post-tests. All values were log-transformed prior to statical analysis to satisfy model assumptions. *Statistically significant difference from the respective control. #Statistically significant difference between exposure groups

The high effects on CYP expression induced by the DEP_B0_ and DEP_B7_ samples seen in Figs. 4 and 5 suggest that the responses to these particles may in part be mediated by AhR. To directly assess the involvement of this pathway, the cells were treated with 10 µM of the AhR-specific inhibitor CH223191 for 30 min prior to particle exposure. This set of experiments was only performed using the DEP_B0_ sample as it produced the strongest effects in combination with α-quartz. The concentration of 25 µg/mL DEP_B0_ and 100 µg/mL α-quartz were chosen as these concentrations were the lowest to cause significant increase in both CXCL8 and IL-1β release compared with the individual particle samples when applied in combination. Pre-treatment with CH223191 caused a statistically significant decrease in CXCL8 and IL-1β secretion induced by the combination of DEP_B0_ and α-quartz, and CXCL8 induced by DEP_B0_ alone, confirming that the effects of the experimental treatments involving DEP_B0_ are partially dependent on the AhR pathway (Fig. 6).

### Soluble organic components of DEP contribute to the increased effects of combined exposure to DEP and α-quartz

**Fig. 7:**
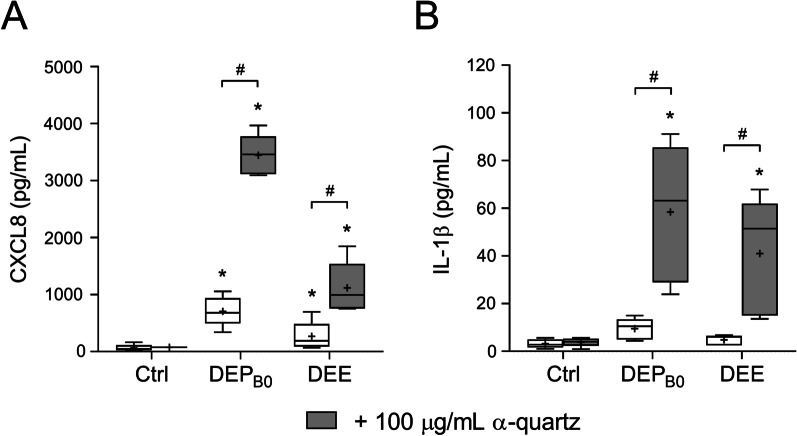
Cytokine responses after exposure to α-quartz in combination with DEP_B0_ or organic extracts of DEP_B0_. HBEC3-KT cells were exposed for 24 h to 25 μg/mL (2.6 μg/cm^2^) of DEP_B0_ and corresponding concentrations of DEP_B0_ extracts (DEE), alone or in combination with 100 μg/mL (10.5 μg/cm^2^) α-quartz. The release of CXCL8 (**A**) and IL-1β (**B**) in the cell culture supernatants was measured using ELISA. Results are presented as boxplots of 5 independent experiments (Box: 25th–75th percentile, whiskers: minimum and maximum values, line: median, +: mean). Statistically significant differences were determined using two-way repeated measures ANOVA followed by Dunnett’s and Šídák's post-tests. All values were log-transformed prior to statistical analysis to satisfy model assumptions. *Statistically significant difference from the respective control. #Statistically significant difference between exposure groups

Studies suggest that much of the effects of DEP can be attributed to soluble organic chemicals [30, 31, 60, 61]. To determine whether soluble particle constituents were responsible for the effects observed in the present study, HBEC3-KT cells were exposed to corresponding concentrations of DEP_B0_ or lipophilic organic extracts of DEP_B0_ (DEE) alone or in combination with α-quartz. None of the experimental treatments caused any significant change in cell viability (Data not shown). Both DEP_B0_ and DEE induced increased secretion of CXCL8 compared with unexposed controls (Fig. 7a). When applied in combination with 100 µg/mL α-quartz, CXCL8 secretion increased significantly compared with exposure to DEP_B0_ and DEE alone, even though α-quartz was largely ineffective by itself in this set of experiments (Fig. 7a). While DEP_B0_ and DEE failed to induce a statistically significant increase in IL-1β secretion when applied alone, IL-1β secretion increased significantly when cells were exposed in combination with α-quartz (Fig. 7b). Interestingly, the relative increase in CXCL8 and IL-1β release from exposure to DEP_B0_ and DEE alone compared to combined exposure with α-quartz was similar for the native particles and the extracts. Taken together, the results suggest that much of the effects of combined exposure to DEP and α-quartz may be attributed to lipophilic organic chemicals.

### Stone particles of different mineral composition potentiate the effects of DEP on cytokine release and expression of genes related to inflammation and redox responses

**Fig. 8:**
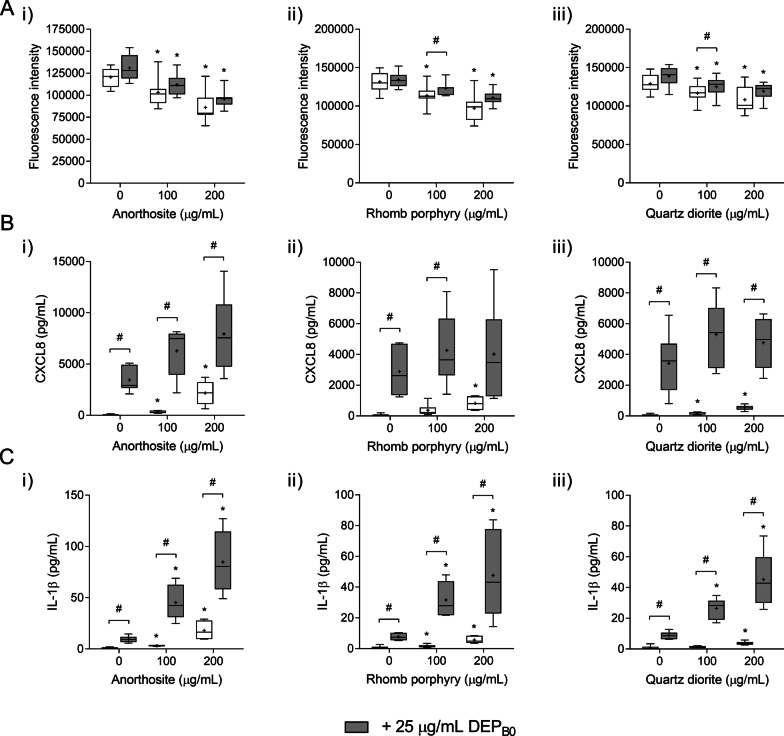
Cytotoxicity and cytokine release induced by combined exposure to stone particles and DEP_B0_. HBEC3-KT cells were exposed for 24 h to 25 μg/mL (2.6 μg/cm^2^) of DEP_B0_ alone or in combination with 100–200 μg/mL (10.5–21.1 μg/cm^2^) anorthosite (i), rhomb porphyry (ii) or quartz diorite (iii) particles. Cell viability (**A**) was determined using alamarBlue assay while the release of CXCL8 (**B**) and IL-1β (**C**) in the cell culture supernatants was measured using ELISA. Results are presented as boxplots of 6–7 independent experiments (Box: 25th–75th percentile, whiskers: minimum and maximum values, line: median, +: mean). Statistically significant differences were determined using two-way repeated measures ANOVA followed by Dunnett’s and Šídák's post-tests. Values not adhering to model assumptions were log-transformed before statistical analysis. *Statistically significant difference from the respective control at 0 μg/mL mineral particles. #Statistically significant difference between exposure groups

Finally, the combinative effects of DEP and minerals in HBEC3-KT cells were assessed using stone samples of mixed mineral composition. Again, only the DEP_B0_ sample was used in this set of experiments as it produced the strongest effects on cytokine release in combination with α-quartz. Samples of anorthosite, rhomb porphyry and quartz diorite particles (< 10 μm) were derived from crushed stone material and consist of a mixture of different minerals, including feldspar, quartz, epidote, hornblende, calcite, chlorite and muscovite. The characteristics of the stone particle samples and their ability to induce cytotoxicity and pro-inflammatory cytokines have been described in a previous study using the same cell model as in the present study [48]. Moreover, the mineral composition of the samples is included in the online supplement of the current study (Additional file 3: Table S1). Due to the lower toxicity of these particle samples compared to α-quartz, the concentration-range was extended to 200 µg/mL. For comparison, experiments were also conducted in parallel using α-quartz at the same concentrations (Additional file 4: Figure S3 and Fig. 8).

**Fig. 9:**
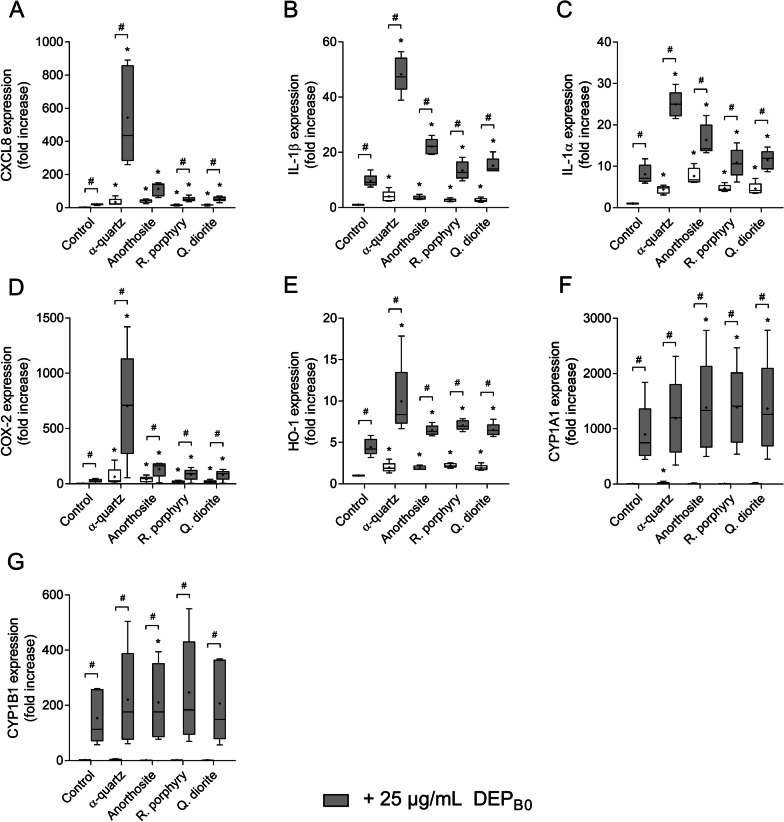
Expression of genes related to inflammation and redox responses after combined exposure to DEP_B0_ and stone particles. HBEC3-KT cells were exposed to 25 μg/mL (2.6 μg/cm^2^) DEP_B0_ alone or in combination with 200 μg/mL (21.1 μg/cm^2^) α-quartz, anorthosite, rhomb porphyry or quartz diorite for 12 h. The expression of CXCL8 (**A**), IL-1β (**B**), IL-1α (**C**), COX-2 (**D**) and HO-1 (**E**), CYP1A1 (**F**) and CYP1B1 (**G**) was determined using qPCR. Results are expressed as fold change compared to unexposed cells and presented as boxplots of 5 independent experiments (Box: 25th–75th percentile, whiskers: minimum and maximum values, line: median, +: mean). Statistically significant differences were determined using two-way repeated measures ANOVA followed by Dunnett’s and Šídák's post-tests and are calculated from normalized CT values. *Statistically significant difference from the respective control at 0 μg/mL mineral particles. #Statistically significant difference between exposure groups

Exposure to anorthosite, rhomb porphyry and quartz diorite particles induced concentration-dependent decreases in cell viability, culminating at approximately 15–30% reduction at the highest concentrations (Fig. 8a). However, no additional decrease was detected when the cells were exposed to stone particle samples in combination with DEP_B0_. All stone particle samples induced statistically significant increases in CXCL8 and IL-1β release compared with unexposed controls, starting at 100 or 200 µg/mL (Fig. 8b, c). Combined exposure with DEP_B0_ tended to increase the release of both cytokines compared with the individual particle samples (Fig. 8b, c). For IL-1β, the response induced by combined exposure was significantly greater than the response induced by DEP_B0_ alone for all stone particle samples (Fig. 8c). All stone particle samples caused significant increases in the expression of CXCL8, IL-1β, IL-1α, COX-2 and HO-1 (Fig. 9a–e). Moreover, combined exposure with DEP_B0_ induced a further increase in gene expression, exceeding the effects of exposure to the individual particulate samples (Fig. 9a–e). Although the effects of the stone particle samples on cytokine release and gene expression were lower than observed for α-quartz at the same concentrations (Additional file 5: Figure S3 and Fig. 9), these results clearly show that the effects of combined exposure to DEP and mineral particles on pro-inflammatory responses in HBEC3-KT cells also extend to mineral samples composed primarily of minerals other than α-quartz.

**Fig. 10:**
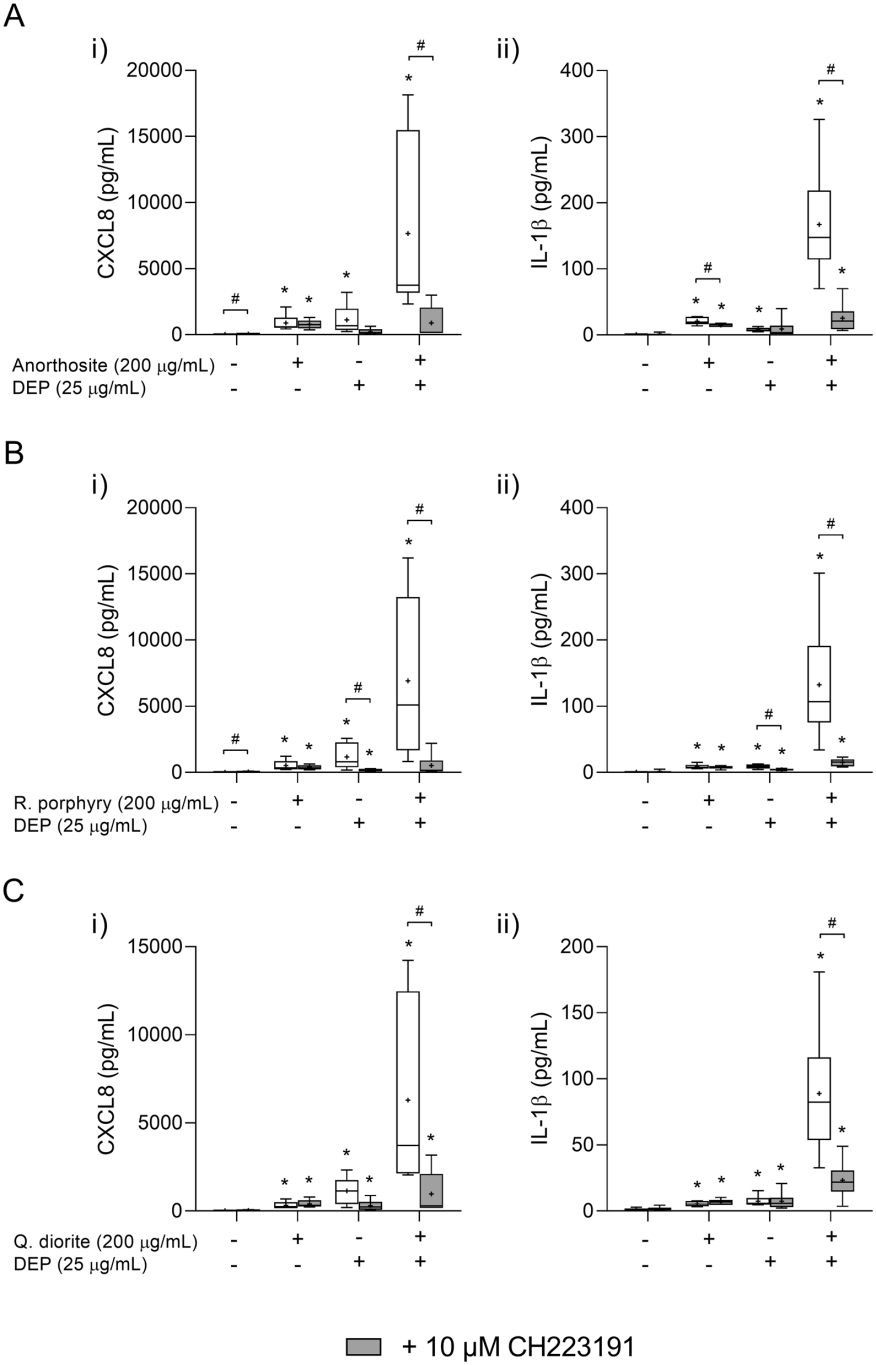
The effects of AhR-inhibition on the cytokine responses induced by combined exposure to DEP_B0_ and stone particles. HBEC3-KT cells were exposed to the AhR-specific inhibitor CH223191 (10 μM) for 30 min prior to 24 h exposure to 25 μg/mL (2.6 μg/cm^2^) DEP_B0_ and 200 μg/mL (21.1 μg/cm^2^) anorthosite (**A**), rhomb porphyry (**B**) and quartz diorite (**C**). The release of CXCL8 (i) and IL-1β (ii) in the cell culture supernatants was measured using ELISA. Results are presented as boxplots of 5 independent experiments (Box: 25th–75th percentile, whiskers: minimum and maximum values, line: median, +: mean). Statistically significant differences were determined using two-way repeated measures ANOVA followed by Dunnett’s and Šídák's post-tests. All values were log-transformed prior to statical analysis to satisfy model assumptions. *Statistically significant difference from the respective control. #Statistically significant difference between exposure groups

None of the stone particle samples significantly altered the expression of CYP1A1 and CYP1B1 by themselves, although they seemed to potentiate the effects of DEP_B0_ on CYP expression when administered in combination (Fig. 9f, g). Combined exposure caused a significant increase in CYP1A1 compared with DEP_B0_ alone for all stone particle samples, and a significant increase in CYP1B1 for anorthosite (Fig. 9f, g). Pre-incubation with the AhR antagonist CH223191 caused a strong inhibition of cytokine release induced by the combined exposure to DEP_B0_ and anorthosite, rhomb porphyry or quartz diorite (Fig. 10), suggesting that these combinatory effects were almost completely dependent on AhR signalling, as also observed with α-quartz (Fig. 6).

## Discussion

PM in ambient air and occupational settings often consists of a mixture of particles from multiple sources. While the effects of PM mixtures have been the subject of numerous experimental studies, few have assessed the effects of combined exposure to particles from different sources in a systematic way. In the present study, the effects of DEP and mineral particles, two common PM constituents, were assessed in human bronchial epithelial cells after exposure to the particle samples alone and in combination. The results show that combined exposure to DEP and mineral particles significantly increased the particle-induced pro-inflammatory responses in HBEC3-KT cells, as compared to their individual effects. This suggests that combinatory effects of exposure to particles and particle components from different sources may contribute to or exacerbate the adverse health effects triggered by complex PM. Furthermore, the present findings suggest a central role for lipophilic organic chemicals in these effects.

In the present study, combined exposure caused increases in cellular responses that were greater than exposure to either particle sample alone. This included increased secretion of the pro-inflammatory cytokines CXCL8 and IL-1β, and increased gene expression of CXCL8, IL-1β, IL-1α, COX-2 and HO-1. The effects were detected for both DEP_B0_ and for DEP_B7_ in combination with α-quartz, and for DEP_B0_ in combination with anorthosite, rhomb porphyry and quartz diorite particles, suggesting that the enhanced effects can be induced by several exposure scenarios. Moreover, in several cases the effect of combined exposure far surpassed the effects of the particle samples alone, which was particularly prominent for the release of CXCL8 and IL-1β and for the expression of CXCL8 and COX-2. However, more in-depth studies will be needed to confirm whether the effects of combined exposure are additive or synergistic [62, 63].

Few other studies have assessed the effects of combined exposure to minerals and combustion particles in experimental systems [64–66]. Tomašek et al. [66] exposed an alveolar 3D co-culture at air–liquid interface (ALI) and pseudo-ALI to volcanic ash particles in combination with the National Institute of Standards and Technology (NIST) standard reference material (SRM) 2975, a DEP derived from an industrial forklift. However, the results were mostly negative, and only a small non-significant increase in tumor necrosis factor (TNF)α and IL-1β secretion was observed after combined exposure compared to the particle samples alone [66]. Similar results were reported in the same cell model exposed to volcanic ash and complete gasoline exhaust, which produced no significant change in cytotoxicity or markers of oxidative stress and inflammation [65]. In a study by Farris et al. [64], the effects of SRM 2975 and quartz were assessed in rats after intratracheal instillation of doses relevant to workers in hydraulic fracturing operations. While combined exposure modified the effects on lung injury and inflammation, no consistent increase in biological endpoints were detected across doses and time points, and DEP tended to decrease the quartz-induced levels of several inflammatory markers in the lung when administered together [64]. Nevertheless, combined exposure induced an increase in the number of neutrophils, which persisted up to 1 month after exposure, indicating increased lung inflammation. The NIST SRM 2975 used in these studies is a sample originally intended as a standard for chemical analysis but is frequently used in toxicological studies. Notably, this DEP sample was collected at high temperatures, which limits the condensation of PAHs and other semi-volatiles [67]. Consequently, SRM 2975 is essentially a denuded DEP with considerably lower levels of PAHs and other organic constituents than the samples used in the present study, as well as other DEP samples tested [21, 30, 53, 67, 68]. Thus, it seems likely that the more limited combinatory effects observed in Tomašek et al. [66] and Farris et al. [64] could be explained by the lack of organic material in SRM 2975. Several studies suggest that lipophilic particle constituents are important drivers of the pro-inflammatory effects of DEP [30, 31, 60, 61]. This is also exemplified in the present study where lipophilic organic extracts of DEP_B0_ (DEE) induced similar responses in combination with α-quartz as the native DEP_B0_ particles. However, it should be noted that the pro-inflammatory potential of DEP does not necessarily correspond to the content of organic particle constituents, suggesting that the specific composition rather than the total organic content may be of importance [69, 70]. Moreover, SRM 2975 appears to induce biological effects through other particle constituents, such as more polar compounds, metals or the carbon particle core [71–74].

In the present study, the pro-inflammatory effects of DEP alone and in combination with α-quartz varied between the DEP samples generated from different fuels. DEP_B0_ induced the highest levels of CXCL8 secretion followed by DEP_B7_, while DEP_B20_ induced the lowest amount. The IL-1β response was more similar between the DEP samples, although there was a tendency for lower response in cells exposed to DEP_B20_. A similar order of potency was observed when cells were exposed to DEP and α-quartz in combination, with DEP_B0_ inducing the highest CXCL8 and IL-1β responses. In addition, the increase in both cytokines was higher and more consistent with DEP_B7_ compared with DEP_B20_, reaching statistical significance compared with DEP alone. While DEP_B0_ and DEP_B7_ induced comparable levels of CXCL8, IL-1β, IL-1α, HO-1 and COX-2 expression, DEP_B0_ tended to induce somewhat higher responses in combination with α-quartz. As reviewed by Moller et al. [75], previous studies assessing the pro-inflammatory effects of emissions from biodiesel and conventional petroleum diesel in experimental animals or in lung epithelial cells in vitro have shown inconsistent results, with some studies reporting higher toxicity of biodiesel emissions than conventional diesel fuel, and others reporting similar or lower effects. The more pronounced effects of the DEP_B0_ and DEP_B7_ samples compared with DEP_B20_ observed in the present study might indicate that increasing the biodiesel content of the fuel may decrease the pro-inflammatory effects of the resulting particles. However, this should be interpreted with some caution as the differences between the DEP_B0_ and the DEP_B7_ and DEP_B20_ samples could partly be due to differences in engine technology and sampling conditions, rather than the fuel composition.

The increased pro-inflammatory effects of combined exposure were also observed using particle samples of mixed mineral composition derived from crushed stone material. As for α-quartz, exposure to anorthosite, rhomb porphyry or quartz diorite particles in combination with DEP_B0_ induced significant increases in the secretion of pro-inflammatory cytokines and expression of genes related to inflammation and redox responses compared with exposure to the individual particle samples. The potency of the mineral samples in combination with DEP_B0_ was mostly in line with the order of potency reported for the individual samples in previous studies using the same cell model, with α-quartz being the most potent followed by anorthosite, while rhomb porphyry and quartz diorite induced lower and similar levels [48]. Considering that the anorthosite does not contain any quartz (Additional file 3: Table S1) [48], these results clearly show that the increased effects of combined exposure cannot be explained by quartz content alone, suggesting that other mineral components are important. Consequently, this means that minerals not commonly considered particularly toxic may increase the effects of DEP when administered in combination.

The AhR pathway is highly expressed in lung tissue and can be activated by components of particulate air pollution, such as PAHs [58, 59]. In the present study, robust increases in expression of the AhR-target genes CYP1A1 and CYP1B1 were detected after exposure to DEP, both alone and in combination with mineral particles. Moreover, exposure to the AhR-specific inhibitor CH223191 prior to particle exposure significantly attenuated the particle-induced cytokine responses, further suggesting a role of the AhR pathway in the pro-inflammatory effects of DEP and minerals in HBEC3-KT. This is in line with previous studies from our group and others showing that AhR is involved in the pro-inflammatory responses induced by DEP or DEP extractable organic chemicals in airway epithelial cells and vascular endothelial cells [30, 53, 76]. AhR is known to regulate a number of cytokine genes directly through conventional xenobiotic response elements (XRE) in their promotor region or through interactions with other transcription factors such as nuclear factor kappa B (NF-κB) [76–78]. Canonical ligands for AhR include PAHs and halogenated aromatic hydrocarbons, many of which are abundant in combustion-derived PM [58, 59]. In the present study, lipophilic organic extracts of DEP_B0_ showed enhanced effects on cytokine secretion similar to the native particles when administered together with quartz, suggesting a central role for soluble organic chemicals. Moreover, previous chemical characterization of the DEP samples suggests that the content of PAHs corresponds to the order of potency observed in the present study, with DEP_B0_ containing the largest amount, followed by DEP_B7_ and DEP_B20_ [21, 30, 53]. This is in line with the results of Brinchmann et al. [30] who showed that lipophilic extracts of DEP induced IL-1β, CXCL8, COX-2 and HO-1 expression in human endothelial cells through mechanisms partly dependent on AhR. However, it should be noted that the residual particles after extraction of organic chemicals have been reported to induce CYP1A1 expression to a larger extent than organic extracts [31], suggesting that the carbon particle core or less extractable components may also contribute to the effects of DEP. In addition to their capacity as AhR agonists, metabolism of PAHs, for instance by CYP enzymes, may cause the formation of electrophilic and redox-active metabolites, such as diol epoxides, radical cations and quinones, which may damage cellular macromolecules [79]. In the present study, the expression of HO-1, an enzyme linked to the redox-sensitive pathway nuclear factor erythroid 2-related factor 2 (Nrf2) [57], was elevated in cells exposed to DEP and minerals in combination, suggesting a possible role for formation of reactive oxygen species (ROS) and oxidative stress. However, previous studies from our group suggest that DEP activates AhR and pro-inflammatory responses at lower concentrations and occur in the absence of effects on HO-1 expression [30, 53], suggesting that the effects on oxidative stress could be a high dose phenomenon rather than an underlying mechanism. Taken together, the results of the present study suggest that lipophilic organic chemicals in DEP are involved in the effects of combined exposure, which are mediated through activation of the AhR signalling pathway.

The possibility of altered effects from combined exposure to pollutants may be of concern from a public and occupational health perspective, especially if the effects are increased, the threshold dose for adverse effects is lowered, or if the combination causes adverse effects that are not caused by the individual compounds. In the present study, the in vitro pro-inflammatory effects of combined exposure to DEP and mineral particles surpassed the effects of the individual particulates, suggesting that combined exposure may be more detrimental to human health. Consequently, our findings indicate that the potential benefits of phasing out or eliminating specific sources of PM, such as diesel fuel combustion, could be greater than anticipated based on studies of the individual components, and possibly exceed the effects of more general measures to reduce overall emissions of total PM. Thus, characterisation of possible combinatory effects between particles from different sources could aid policymakers in determining more efficient mitigating measures targeting the pollutants that contribute the most to adverse effects.

There are some limitations with the current study that should be mentioned. First, the current study uses a simplified in vitro model, consisting of monocultures of lung epithelial cells exposed under submerged conditions, and does not mimic the complexity of in vivo exposure. Thus, care must be taken when extrapolating these results to human exposure scenarios. Recent advances in in vitro model systems include more complex 3D cell models consisting of multiple cell types and exposure at ALI, which offer a more relevant model system for inhalation exposure [80–82]. Second, the concentrations used in this study are quite high. Li et al. [83] estimated that 24 h exposure to 79 μg/m^3^ PM_2.5_ in urban air could translate to 204 μg/cm^2^ in the upper airways, 2.3 μg/cm^2^ in the tracheobronchial region, and 0.05 μg/cm^2^ in the alveolar region. In comparison, the concentrations used in the present study were in the range of 2.6–21.1 μg/cm^2^. However, it’s important to note that exposure to DEP and minerals is also relevant for exposure in occupational settings, such as mining and tunnel construction, where the particle concentrations may be orders of magnitude higher than in ambient air. For reference, the US OSHA regulates most mineral dust as respiratory dust (PM_5_) in general, with an occupational exposure limit of 5 mg/m^3^ as an 8-h time weighted average [84]. Nevertheless, the concentrations used in the present study are likely higher than concentrations that could be expected to occur in most real-life exposure scenarios, which should be considered when interpreting the results of the study. Thus, future studies should focus on verifying the findings in more advanced model systems and more relevant exposure concentrations. However, it should be noted that Farris et al. [64] found that combined exposure to DEP and α-quartz induced significant increases in neutrophil influx in rat lungs at doses they estimated to be relevant for high exposure in hydraulic fracturing operations, suggesting that the effects observed in the current study are not just a high dose phenomenon or an artefact of the cell model system.

## Conclusions

The present study indicates that combined exposure to DEP and mineral particles, two common constituents of PM in ambient air and occupational settings, induces pro-inflammatory responses in human lung epithelial cells that often greatly exceed the effect of either sample alone. The effects were observed both with DEP generated using an older diesel engine running on petroleum diesel and DEP from modern biodiesel blends, as well as several mineral samples of different composition, which adds to the robustness of the findings. The responses were partly mediated through an AhR-dependent pathway, and lipophilic organic chemicals in DEP seemed to be major determinants. Taken together, the results of the present study emphasize that combinatory effects of different source-specific components of ambient PM could be an important factor driving the adverse health effects. Importantly, future studies should address whether targeting specific key components of ambient PM may lead to larger health benefits than general measures to reduce the overall emissions.

## Material and methods

### Particle samples

Min-U-Sil 5, referred to as α-quartz in the main body of the text, was purchased from the US Silica company and is a ground silica sample of high purity (Median size 1.6 μm) (US Silica Co., Berkeley Springs, WV, USA). The generation and characterisation of the anorthosite, rhomb porphyry and quartz diorite particle samples have been described in a previous publication [48]. Briefly, the samples were delivered by stone aggregate producers in the size range of 8–16 mm and crushed further in a Los Angeles test machine before extracting the fraction of particles below 10 µm by gravitational settling in water. The mineral composition of the samples was determined using X-ray diffraction (XRD) analysis (Additional file 3: Table S1). The particle size distributions of the samples were similar with > 95% of the particle being below 10 μm and a mean particle size of approximately 4.2 μm, 3.2 μm and 3.5 μm for the anorthosite, rhomb porphyry and quartz diorite samples, respectively. The specific surface area of the particles was 7.2 m^2^/g for the anorthosite and rhomb porphyry samples, and 5.1 m^2^/g for the quartz diorite sample [48]. The process of collection and characterization of the DEP samples has also been described in previous publications [21, 53]. Briefly, DEP_B0_ was collected by scraping deposited particles from the wall of the main exhaust pipe of a diesel engine (Deutz, 4-cylinder, 2.2 L, 500 RPM) running on gas oil (Petroplus Refining Teesside Ltd., United Kingdom), and was provided by Dr. Flemming R. Cassee (RIVM, Netherland). The DEP_B7_ and DEP_B20_ samples were collected from a Euro 5 diesel engine without diesel particle filter (Common Rail 3^rd^ generation injection system, 1.2 L, 1248 cm^3^, max power 75 bhp, max torque 190 nM, production year 2014) running on 1st generation biodiesel fuel containing either 7% or 20% fatty acid methyl ester (FAME) in diesel oil, respectively. Both DEP_B7_ and DEP_B20_ were collected directly from the exhaust on polytetrafluoroethylene (PTFE)-coated glass fiber filters (70 mm; Pallflex, Emfab filters, TX40HI20WW). Particles were then gently scraped of the filters for use in cell culture experiments.

### Preparation of particle stock solutions

All particle stock solutions were prepared in cell culture medium and sonicated for 5 min on ice using a Vibra-Cell™ probe sonicator (Sonics & Materials Inc., Newtown, CT, USA) to ensure even suspension of the particles. Due to difficulties in dispersing the DEP particles in cell culture media, the suspended particles were vortexed for 10 min and centrifuged on 1200 × *g* to pellet the particles prior to sonication. The primary particle stock concentration was 2 mg/mL for all samples, which was further diluted in the wells to yield the final concentrations. The mineral particle stocks were prepared in glass vials while the DEP samples were prepared in 1.5 mL Eppendorf tubes.

### Endotoxin concentrations in the particle stocks

Endotoxin in the DEP_B0_, DEP_B7_ and DEP_B20_ particle stock was quantified using the Pierce™ Chromogenic Endotoxin Quant Kit (ThermoFisher Scientific, Waltham, MA, USA) with minor alteration noted in Grytting et al. [48]. Particle suspensions and endotoxin standard solutions were prepared using HyClone™ Water (Fisher Scientific, Waltham, MA, USA) and Lonza Pyrogen-free Test Tubes (Fisher Scientific, Waltham, MA, USA). The concentration of endotoxin in the α-quartz, anorthosite, rhomb porphyry and quartz diorite samples has been reported in a previous study [48].

### Preparation of diesel exhaust particle extract

Soluble organic chemicals were extracted by sequential washing in water under pressure at increasing temperature (25–150 °C), followed by a final washing with methanol (100 °C). The extracted organic material was dissolved in dimethyl sulfoxide (DMSO) for use in cell culture experiments at a concentration corresponding to the mass of DEP used to generate the extracts. Organic chemicals in the extracts were determined using a thermal optical analyser (Sunset Laboratories, Tigard, OR, USA), while individual substances were identified by gas chromatography mass spectrometry using a 6890 Series II Plus GC coupled to a 5975C MS detector (Agilent, Santa Clara, CA). Based on the chemical analysis and an initial screening for effects on cytotoxicity and pro-inflammatory responses (Brinchmann et al. unpublished results), only the methanol soluble, lipophilic fraction was included in the present study.

### Cell cultures and exposure

#### HBEC3-KT

HBEC3-KT cells (ATCC, Manassas, VA, USA) were cultured in LHC-9 medium (Lonza, Basel Switzerland) in collagen-coated T75 flasks and maintained at 37 °C in a humidified atmosphere containing 5% CO_2_. The cells were loosened with trypsin (Sigma-Aldrich, Saint-Louis, MO, USA) and passaged twice every week to maintain proper cell culture conditions. Prior to the experiments, cells were seeded on collagen-coated 12-well cell culture plates at a concentration of 80,000 cells per well (21,000 cells/cm^2^) in 1 mL LHC-9 medium. After 24 h, the LHC-9 medium was replaced, and the cells incubated for an additional 24 h. Then, 24 h prior to exposure, each well was washed with 1 mL phosphate-buffered saline (PBS) before adding 1 mL of DMEM F12 medium (Gibco by Thermofischer, Waltham, MA, USA) supplemented with penicillin–streptomycin (Lonza, Basel, Switzerland), ampicillin (New York, NY, USA) and amphotericin B (Sigma, St. Louis, MA, USA).

#### Exposure regime and sample preparation

HBEC3-KT cells were exposed in 0.4 mL DMEM F12 in 12 well cell culture plates. Particle samples were vortexed vigorously before adding them to the cell culture to ensure even suspension of the particles. Following exposure, the cells were incubated at 37 °C in an atmosphere containing 5% CO_2_ for 2, 6, 12 or 24 h, depending on the experimental design. A microscope image of the cells at the end of an experiment is provided in figure S4 in Additional file 5. To assess the involvement of the AhR pathway, the cells were exposed to 10 μM of the AhR specific inhibitor CH223191 (Sigma-Aldrich, Saint-Louis, MO, USA) for 30 min prior to exposure to the particles. The inhibitor stock was dissolved in DMSO at a concentration of 10 mM and was diluted in cell culture medium to give the final concentration. The final DMSO concentration in the well was kept below 0.1% and was balanced between wells. Following exposure, the cell culture supernatants were transferred to 1.5 mL Eppendorf tubes and centrifuged at 290 × *g* for 10 min to remove cells and debris. The supernatants were then transferred to new Eppendorf tubes and centrifuged for an additional 10 min at 1200 × *g* to remove particles. For mRNA isolation, the cell culture plates were put on ice and each well washed twice with cold PBS (4 °C) after removing the medium. Both the supernatants and the cell culture plates were then stored at − 80 °C awaiting analysis.

### Release of pro-inflammatory cytokines

The concentrations of CXCL8 and IL-1β in the cell culture supernatant were measured using Cytoset (Invitrogen by Thermo Fischer Scientific, Waltham, MA, USA and Novex by Life Technologies Waltham, MA, USA) or Duoset (R&D systems, Minneapolis, MN, USA) enzyme-linked immunosorbent assay (ELISA) kits, respectively, both applied according to the manufacturer’s instructions. Samples were diluted in the recommended assay buffer to stay within the appropriate range of the assay. ELISA was performed in Nunc Maxisorb plates (Thermo Scientific, Waltham, MA, USA). Absorbance was measured using a Sunrise plate reader (Tecan, Männerdorf, Switzerland).

### Cytotoxicity

Cytotoxicity was assessed by the alamarBlue assay (Invitrogen by thermofischer scientific, Waltham, MA, USA) according to the manufacturer’s instructions. The cells were incubated for 60 min with 0.4 mL alamarBlue solution, diluted 1:10 in cell culture medium, at 37 °C in an atmosphere containing 5% CO_2_. Fluorescence was measured using a Clariostar plate reader (BMG LABTECH, Ortenberg, Germany).

### Gene expression analysis

RNA was isolated using the NucleoSpin RNA Plus kit (Macherey-Nage, Düren, Germany) according to the manufacturers’ recommendations. The content and quality of the RNA in each sample were determined using a DS-11 spectrophotometer (Denovix, Wilmington, DE, USA). Next, RNA was reverse transcribed into cDNA using a High Capacity cDNA Reverse Transcription Kit (Applied Biosystems by Thermofischer, Waltham, Ma, USA) and an S-100 thermal cycler (BioRad, Hercules, CA, USA). Gene expression of CXCL8, IL-1α, IL-1β, IL-6, HO-1, COX-2, CYP1A1, CYP1B1 and GAPDH was determined by quantitative real-time polymerase chain reaction (qPCR) using a CFX96 Touch Real-Time PCR Detection System (BioRad, Hercules, CA, USA) with pre-designed TaqMan Gene Expression Assays (CXCL8: Hs00174103_m1, IL-1α: Hs00174092_m1, IL-1β: Hs01555410_m1, COX2: Hs00153133_m1, HO-1: Hs01110250_m1, CYP1A1: Hs01054797_g1, CYP1B1: Hs02382916_s1, GAPDH Hs02758991_g1) and TaqMan Universal PCR Master Mix (Both from Thermofischer, Waltham, MA, USA). The expression of each gene was normalized against the housekeeping gene glyceraldehyde 3-phosphate dehydrogenase (GAPDH) and expressed as fold change compared to unexposed control, calculated according to the ΔΔCt method (ΔCt = Ct[Gene of Interest] − Ct[GAPDH]; ΔΔCt = ΔCt[Treated] − ΔCt[Control]; Fold change = 2[− ΔΔCt]).

### Binding of cytokines to particles

Non-specific binding of cytokines to the DEP_B0_, DEP_B7_ and DEP_B20_ samples was measured as described in previous studies [54]. Solutions of 200 pg/mL CXCL8 and 62.5 pg/mL IL-1β were prepared using the standard from ELISA kits and incubated with 50 μg/mL particles in DMEM cell culture medium in a 24 well cell culture plate at 37 °C in an atmosphere containing 5% CO_2_. After 24 h, the medium was transferred to 1.5 mL Eppendorf tubes and centrifuged for 10 min at 1200 × *g* to pellet the particles. The concentrations of cytokines remaining in the supernatant was measured using ELISA. The amount of cytokine binding by the α-quartz, anorthosite, rhomb porphyry and quartz diorite samples has been published in a recent study [48].

### Statistical analyses

The concentration–response relationships for the different particle samples were analysed by one-way analysis of variance (ANOVA) followed by Dunnett’s post-test for multiple comparisons, while the effect of combined exposure to multiple agents was analysed using two-way ANOVA followed by Dunnett’s, Tukey or Šídák's post-tests, depending on the experimental design. A repeated measures design, in which all values from a single experiment were defined as a subject, was used throughout to account for variation between experiments not related to the experimental treatment. Geisser-Greenhouse correction was applied to account for deviations from sphericity. Log-transformation was used in cases where the data did not meet model assumptions.

## Supplementary Information

**Additional file 1: Figure S1. DEP-induced cytotoxicity and cytokine release.** HBEC3-KT cells were exposed to 12.5–200 μg/mL (1.3–21.1 μg/cm^2^) DEP_B0_ (A), DEP_B7_ (B) or DEP_B20_ (C) for 24 h. The release of CXCL8 (i) and IL-1β (ii) in the cell culture supernatants was measured using ELISA while cell viability (iii) was determined using alamarBlue assay. Results are presented as mean ± SD  (n = 3–7). Statistically significant differences were determined using one-way repeated measures ANOVA followed by Dunnett’s post-test. Values not adhering to model assumptions were log-transformed before statistical analysis. * Statistically significant difference from the respective control.

**Additional file 2: Figure S2. Non-specific binding of cytokines to the DEP samples.** Solutions of 200 pg/mL CXCL8 (A) or 62.5 pg/mL IL-1β (B) were incubated with 50 μg/mL (5.3 μg/cm^2^) DEP_B0_, DEP_B7_ or DEP_B20_ for 24 h. The concentrations cytokines remaining in the medium were measured using ELISA. The experiment was performed in triplicate and is presented as percentage of control (mean ± SD).

**Additional file 3: Table S1.** Mineralogical composition of the anorthosite, rhomb porphyry and quartz diorite samples.

**Additional file 4: Figure S3. Cytotoxicity and cytokine responses after combined exposure to DEP and α-quartz at higher concentrations.** HBEC3-KT cells were exposed to 25 μg/mL (2.6 μg/cm^2^) DEP_B0_ and 100–200 μg/mL (10.5–21.1 μg/cm^2^) α-quartz for 24 h, alone or in combination. Cytotoxicity (A) was measured by AlamarBlue assay, while the release of CXCL8 (B) and IL-1β (C) in the cell culture supernatants was measured using ELISA. Results are presented as boxplots of 6 independent experiments (Box: 25th-75th percentile, whiskers: minimum and maximum values, line: median, +: mean). Statistically significant differences were determined using two-way repeated measures ANOVA followed by Dunnett’s and Tukey post-tests. Values not adhering to model assumptions were log-transformed before statistical analysis. * Statistically significant difference from the respective control at 0 μg/mL α-quartz. # Statistically significant difference between exposure groups.

**Additional file 5: Figure S4. Microscope image of the cells after exposure.** A microscope image of HBEC3-KT cells after exposure to vehicle (A), 100 μg/mL (10.5 μg/cm^2^) α-quartz (B), 50 μg/mL (5.3 μg/cm^2^) DEP_B0_ (C) or 100 μg/mL α-quartz in combination with 50 μg/mL DEP_B0_ (D) for 24 h

## Data Availability

The datasets used in the present study are available from the corresponding author upon reasonable request.

## Abbreviations

AhR: Aryl hydrocarbon receptor
ALI: Air liquid interface
ANOVA: Analysis of variance
COX: Cyclooxygenase
CXCL: Chemokine CXC-motif ligand
CYP: Cytochrome P450
DEE: Diesel exhaust extracts
DEP: Diesel exhaust particles
DMSO: Dimethyl sulfoxide
ELISA: Enzyme-linked immunosorbent assay
FAME: Fatty acid methyl ester
GAPDH: Glyceraldehyde 3-phosphate dehydrogenase
HBEC3-KT: HTERT and CDK4 immortalized human bronchial epithelial cells
HO: Heme oxygenase
IL: Interleukin
NFkB: Nuclear factor kappa B
NIST: National Institute of Science and Technology
Nrf2: Nuclear factor erythroid 2–related factor 2
PAH: Polycyclic aromatic hydrocarbons
PBS: Phosphate-buffered saline
PM: Particulate matter
PTFE: Polytetrafluoroethylene
qPCR: Quantitative real-time polymerase chain reaction
ROS: Reactive oxygen species
SRM: Standard reference material
TNF: Tumor necrosis factor
XRD: X-ray diffraction
XRE: Xenobiotic response elements

## References

[1] Cesaroni G, Forastiere F, Stafoggia M, Andersen ZJ, Badaloni C, Beelen R, Caracciolo B, de Faire U, Erbel R, Eriksen KT (2014). Long term exposure to ambient air pollution and incidence of acute coronary events: prospective cohort study and meta-analysis in 11 European cohorts from the ESCAPE Project. BMJ.

[2] Chen J, Hoek G (2020). Long-term exposure to PM and all-cause and cause-specific mortality: a systematic review and meta-analysis. Environ Int.

[3] Hamra GB, Guha N, Cohen A, Laden F, Raaschou-Nielsen O, Samet JM, Vineis P, Forastiere F, Saldiva P, Yorifuji T (2014). Outdoor particulate matter exposure and lung cancer: a systematic review and meta-analysis. Environ Health Perspect.

[4] Khreis H, Kelly C, Tate J, Parslow R, Lucas K, Nieuwenhuijsen M (2017). Exposure to traffic-related air pollution and risk of development of childhood asthma: a systematic review and meta-analysis. Environ Int.

[5] Shah AS, Lee KK, McAllister DA, Hunter A, Nair H, Whiteley W, Langrish JP, Newby DE, Mills NL (2015). Short term exposure to air pollution and stroke: systematic review and meta-analysis. BMJ.

[6] Zhu R, Chen Y, Wu S, Deng F, Liu Y, Yao W (2013). The relationship between particulate matter (PM10) and hospitalizations and mortality of chronic obstructive pulmonary disease: a meta-analysis. COPD J Chronic Obstr Pulm Dis.

[7] Cullinan P, Muñoz X, Suojalehto H, Agius R, Jindal S, Sigsgaard T, Blomberg A, Charpin D, Annesi-Maesano I, Gulati M (2017). Occupational lung diseases: from old and novel exposures to effective preventive strategies. Lancet Respir Med.

[8] Furusjö E, Sternbeck J, Cousins AP (2007). PM10 source characterization at urban and highway roadside locations. Sci Total Environ.

[9] Qadir R, Schnelle-Kreis J, Abbaszade G, Arteaga-Salas J, Diemer J, Zimmermann R (2014). Spatial and temporal variability of source contributions to ambient PM10 during winter in Augsburg, Germany using organic and inorganic tracers. Chemosphere.

[10] Thurston GD, Ito K, Lall R (2011). A source apportionment of US fine particulate matter air pollution. Atmos Environ.

[11] Bakke B, Ulvestad B, Thomassen Y, Woldbæk T, Ellingsen DG (2014). Characterization of occupational exposure to air contaminants in modern tunnelling operations. Ann Occup Hyg.

[12] Bergdahl IA, Jonsson H, Eriksson K, Damber L, Jarvholm B (2010). Lung cancer and exposure to quartz and diesel exhaust in Swedish iron ore miners with concurrent exposure to radon. Occup Environ Med.

[13] Freund A, Zuckerman N, Baum L, Milek D (2012). Submicron particle monitoring of paving and related road construction operations. J Occup Environ Hyg.

[14] Lu C-F, Yuan X-Y, Li L-Z, Zhou W, Zhao J, Wang Y-M, Peng S-Q (2015). Combined exposure to nano-silica and lead induced potentiation of oxidative stress and DNA damage in human lung epithelial cells. Ecotoxicol Environ Saf.

[15] Steerenberg P, Withagen C, Van Dalen W, Dormans J, Cassee F, Heisterkamp S, Van Loveren H (2004). Adjuvant activity of ambient particulate matter of different sites, sizes, and seasons in a respiratory allergy mouse model. Toxicol Appl Pharmacol.

[16] Wang G, Zhao J, Jiang R, Song W (2015). Rat lung response to ozone and fine particulate matter (PM2. 5) exposures. Environ Toxicol.

[17] Wang Z, Zhao J, Wang T, Du X, Xie J (2019). Fine-particulate matter aggravates cigarette smoke extract–induced airway inflammation via Wnt5a–ERK pathway in COPD. Int J Chron Obstruct Pulmon Dis.

[18] Wong EM, Walby WF, Wilson DW, Tablin F, Schelegle ES (2018). Ultrafine particulate matter combined with ozone exacerbates lung injury in mature adult rats with cardiovascular disease. Toxicol Sci.

[19] Pronk A, Coble J, Stewart PA (2009). Occupational exposure to diesel engine exhaust: a literature review. J Expo Sci Environ Epidemiol.

[20] Bendtsen KM, Gren L, Malmborg VB, Shukla PC, Tunér M, Essig YJ, Krais AM, Clausen PA, Berthing T, Loeschner K (2020). Particle characterization and toxicity in C57BL/6 mice following instillation of five different diesel exhaust particles designed to differ in physicochemical properties. Part Fibre Toxicol.

[21] Lankoff A, Brzoska K, Czarnocka J, Kowalska M, Lisowska H, Mruk R, Øvrevik J, Wegierek-Ciuk A, Zuberek M, Kruszewski M (2017). A comparative analysis of in vitro toxicity of diesel exhaust particles from combustion of 1st-and 2nd-generation biodiesel fuels in relation to their physicochemical properties—the FuelHealth project. Environ Sci Pollut Res.

[22] Maricq MM (2007). Chemical characterization of particulate emissions from diesel engines: a review. J Aerosol Sci.

[23] Hussain S, Laumbach R, Coleman J, Youssef H, Kelly-McNeil K, Ohman-Strickland P, Zhang J, Kipen H (2012). Controlled exposure to diesel exhaust causes increased nitrite in exhaled breath condensate among subjects with asthma. J Occup Environ Med.

[24] Salvi S, Blomberg A, Rudell B, Kelly F, Sandstrom T, Holgate ST, Frew A (1999). Acute inflammatory responses in the airways and peripheral blood after short-term exposure to diesel exhaust in healthy human volunteers. Am J Respir Crit Care Med.

[25] Stenfors N, Nordenhall C, Salvi SS, Mudway I, Soderberg M, Blomberg A, Helleday R, Levin JO, Holgate ST, Kelly FJ (2004). Different airway inflammatory responses in asthmatic and healthy humans exposed to diesel. Eur Respir J.

[26] Törnqvist H, Mills NL, Gonzalez M, Miller MR, Robinson SD, Megson IL, MacNee W, Donaldson K, Soderberg S, Newby DE (2007). Persistent endothelial dysfunction in humans after diesel exhaust inhalation. Am J Respir Crit Care Med.

[27] Fizeșan I, Chary A, Cambier S, Moschini E, Serchi T, Nelissen I, Kiss B, Pop A, Loghin F, Gutleb AC (2018). Responsiveness assessment of a 3D tetra-culture alveolar model exposed to diesel exhaust particulate matter. Toxicol In Vitro.

[28] Robertson S, Gray GA, Duffin R, McLean SG, Shaw CA, Hadoke PW, Newby DE, Miller MR (2012). Diesel exhaust particulate induces pulmonary and systemic inflammation in rats without impairing endothelial function ex vivo or in vivo. Part Fibre Toxicol.

[29] IARC (2014). Diesel and gasoline engine exhausts and some nitroarenes. IARC monographs on the evaluation of carcinogenic risks to humans.

[30] Brinchmann BC, Skuland T, Rambøl MH, Szoke K, Brinchmann JE, Gutleb AC, Moschini E, Kubátová A, Kukowski K, Le Ferrec E (2018). Lipophilic components of diesel exhaust particles induce pro-inflammatory responses in human endothelial cells through AhR dependent pathway (s). Part Fibre Toxicol.

[31] Totlandsdal AI, Herseth JI, Bølling AK, Kubátová A, Braun A, Cochran RE, Refsnes M, Øvrevik J, Låg M (2012). Differential effects of the particle core and organic extract of diesel exhaust particles. Toxicol Lett.

[32] Esswein EJ, Breitenstein M, Snawder J, Kiefer M, Sieber WK (2013). Occupational exposures to respirable crystalline silica during hydraulic fracturing. J Occup Environ Hyg.

[33] Radnoff D, Todor MS, Beach J (2014). Occupational exposure to crystalline silica at Alberta work sites. J Occup Environ Hyg.

[34] Semple S, Green DA, McAlpine G, Cowie H, Seaton A (2008). Exposure to particulate matter on an Indian stone-crushing site. Occup Environ Med.

[35] Amato F, Pandolfi M, Moreno T, Furger M, Pey J, Alastuey A, Bukowiecki N, Prevot ASH, Baltensperger U, Querol X (2011). Sources and variability of inhalable road dust particles in three European cities. Atmos Environ.

[36] Kupiainen K, Ritola R, Stojiljkovic A, Pirjola L, Malinen A, Niemi J (2016). Contribution of mineral dust sources to street side ambient and suspension PM10 samples. Atmos Environ.

[37] Terzi E, Argyropoulos G, Bougatioti A, Mihalopoulos N, Nikolaou K, Samara C (2010). Chemical composition and mass closure of ambient PM10 at urban sites. Atmos Environ.

[38] Gustafsson M, Blomqvist G, Gudmundsson A, Dahl A, Swietlicki E, Bohgard M, Lindbom J, Ljungman A (2008). Properties and toxicological effects of particles from the interaction between tyres, road pavement and winter traction material. Sci Total Environ.

[39] Penkała M, Ogrodnik P, Rogula-Kozłowska W (2018). Particulate matter from the road surface abrasion as a problem of non-exhaust emission control. Environments.

[40] Hussein T, Johansson C, Karlsson H, Hansson H-C (2008). Factors affecting non-tailpipe aerosol particle emissions from paved roads: on-road measurements in Stockholm, Sweden. Atmos Environ.

[41] Candeias C, Vicente E, Tome M, Rocha F, Avila P, Alves C (2020). Geochemical, mineralogical and morphological characterisation of road dust and associated health risks. Int J Environ Res Public Health.

[42] Gunawardana C, Goonetilleke A, Egodawatta P, Dawes L, Kokot S (2012). Source characterisation of road dust based on chemical and mineralogical composition. Chemosphere.

[43] Lu S, Luan Q, Jiao Z, Wu M, Li Z, Shao L, Wang F (2007). Mineralogy of Inhalable Particulate Matter (PM10) in the atmosphere of Beijing, China. Water Air Soil Pollut.

[44] Calvert G, Rice F, Boiano J, Sheehy J, Sanderson W (2003). Occupational silica exposure and risk of various diseases: an analysis using death certificates from 27 states of the United States. Occup Environ Med.

[45] Liu Y, Steenland K, Rong Y, Hnizdo E, Huang X, Zhang H, Shi T, Sun Y, Wu T, Chen W (2013). Exposure-response analysis and risk assessment for lung cancer in relationship to silica exposure: a 44-year cohort study of 34,018 workers. Am J Epidemiol.

[46] Steenland K, Mannetje A, Boffetta P, Stayner L, Attfield M, Chen J, Dosemeci M, DeKlerk N, Hnizdo E, Koskela R (2001). Pooled exposure–response analyses and risk assessment for lung cancer in 10 cohorts of silica-exposed workers: an IARC multicentre study. Cancer Causes Control.

[47] Becher R, Hetland RB, Refsnes M, Dahl JE, Dahlman HJ, Schwarze PE (2001). Rat lung inflammatory responses after in vivo and in vitro exposure to various stone particles. Inhal Toxicol.

[48] Grytting VS, Refsnes M, Øvrevik J, Halle MS, Schönenberger J, van der Lelij R, Snilsberg B, Skuland T, Blom R, Låg M (2021). Respirable stone particles differ in their ability to induce cytotoxicity and pro-inflammatory responses in cell models of the human airways. Part Fibre Toxicol.

[49] Hetland RB, Refsnes M, Myran T, Johansen BV, Uthus N, Schwarze PE (2000). Mineral and/or metal content as critical determinants of particle-induced release of IL-6 and IL-8 from A549 cells. J Toxicol Environ Health A.

[50] Øvrevik J, Myran T, Refsnes M, Låg M, Becher R, Hetland RB, Schwarze PE (2005). Mineral particles of varying composition induce differential chemokine release from epithelial lung cells: importance of physico-chemical characteristics. Ann Occup Hyg.

[51] Lindbom J, Gustafsson M, Blomqvist G, Dahl A, Gudmundsson A, Swietlicki E, Ljungman AG (2006). Exposure to wear particles generated from studded tires and pavement induces inflammatory cytokine release from human macrophages. Chem Res Toxicol.

[52] Lindbom J, Gustafsson M, Blomqvist G, Dahl A, Gudmundsson A, Swietlicki E, Ljungman AG (2007). Wear particles generated from studded tires and pavement induces inflammatory reactions in mouse macrophage cells. Chem Res Toxicol.

[53] Totlandsdal AI, Cassee FR, Schwarze P, Refsnes M, Lag M (2010). Diesel exhaust particles induce CYP1A1 and pro-inflammatory responses via differential pathways in human bronchial epithelial cells. Part Fibre Toxicol.

[54] Kocbach A, Totlandsdal A, Låg M, Refsnes M, Schwarze P (2008). Differential binding of cytokines to environmentally relevant particles: a possible source for misinterpretation of in vitro results?. Toxicol Lett.

[55] Turner MD, Nedjai B, Hurst T, Pennington DJ (2014). Cytokines and chemokines: at the crossroads of cell signalling and inflammatory disease. BBA-Mol Cell Res.

[56] Kawahara K, Hohjoh H, Inazumi T, Tsuchiya S, Sugimoto Y (2015). Prostaglandin E2-induced inflammation: Relevance of prostaglandin E receptors. Biochim Biophys Acta.

[57] Ahmed SM, Luo L, Namani A, Wang XJ, Tang X (2017). Nrf2 signaling pathway: pivotal roles in inflammation. Biochim Biophys Acta Mol Basis Dis.

[58] Esser C, Rannug A (2015). The aryl hydrocarbon receptor in barrier organ physiology, immunology, and toxicology. Pharmacol Rev.

[59] Vogel CFA, Van Winkle LS, Esser C, Haarmann-Stemmann T (2020). The aryl hydrocarbon receptor as a target of environmental stressors—implications for pollution mediated stress and inflammatory responses. Redox Biol.

[60] Bonvallot V, Baeza-Squiban A, Baulig A, Brulant S, Boland S, Muzeau F, Barouki R, Marano F (2001). Organic compounds from diesel exhaust particles elicit a proinflammatory response in human airway epithelial cells and induce cytochrome p450 1A1 expression. Am J Respir Cell Mol Biol.

[61] Kawasaki S, Takizawa H, Takami K, Desaki M, Okazaki H, Kasama T, Kobayashi K, Yamamoto K, Nakahara K, Tanaka M (2001). Benzene-extracted components are important for the major activity of diesel exhaust particles: effect on interleukin-8 gene expression in human bronchial epithelial cells. Am J Respir Cell Mol Biol.

[62] Huang RY, Pei L, Liu Q, Chen S, Dou H, Shu G, Yuan ZX, Lin J, Peng G, Zhang W (2019). Isobologram analysis: a comprehensive review of methodology and current research. Front Pharmacol.

[63] Tallarida RJ (2011). Quantitative methods for assessing drug synergism. Genes Cancer.

[64] Farris BY, Antonini JM, Fedan JS, Mercer RR, Roach KA, Chen BT, Schwegler-Berry D, Kashon ML, Barger MW, Roberts JR (2017). Pulmonary toxicity following acute coexposures to diesel particulate matter and alpha-quartz crystalline silica in the Sprague-Dawley rat. Inhal Toxicol.

[65] Tomašek I, Horwell CJ, Bisig C, Damby DE, Comte P, Czerwinski J, Petri-Fink A, Clift MJD, Drasler B, Rothen-Rutishauser B (2018). Respiratory hazard assessment of combined exposure to complete gasoline exhaust and respirable volcanic ash in a multicellular human lung model at the air–liquid interface. Environ Pollut.

[66] Tomašek I, Horwell CJ, Damby DE, Barosova H, Geers C, Petri-Fink A, Rothen-Rutishauser B, Clift MJ (2016). Combined exposure of diesel exhaust particles and respirable Soufriere Hills volcanic ash causes a (pro-)inflammatory response in an in vitro multicellular epithelial tissue barrier model. Part Fibre Toxicol.

[67] Singh P, DeMarini DM, Dick CA, Tabor DG, Ryan JV, Linak WP, Kobayashi T, Gilmour MI (2004). Sample characterization of automobile and forklift diesel exhaust particles and comparative pulmonary toxicity in mice. Environ Health Perspect.

[68] Stevens T, Cho SH, Linak WP, Gilmour MI (2009). Differential potentiation of allergic lung disease in mice exposed to chemically distinct diesel samples. Toxicol Sci.

[69] Jaramillo IC, Sturrock A, Ghiassi H, Woller DJ, Deering-Rice CE, Lighty JS, Paine R, Reilly C, Kelly KE (2017). Effects of fuel components and combustion particle physicochemical properties on toxicological responses of lung cells. J Environ Sci Health Part A.

[70] Tal TL, Simmons SO, Silbajoris R, Dailey L, Cho SH, Ramabhadran R, Linak W, Reed W, Bromberg PA, Samet JM (2010). Differential transcriptional regulation of IL-8 expression by human airway epithelial cells exposed to diesel exhaust particles. Toxicol Appl Pharmacol.

[71] DeMarini DM, Brooks LR, Warren SH, Kobayashi T, Gilmour MI, Singh P (2004). Bioassay-directed fractionation and salmonella mutagenicity of automobile and forklift diesel exhaust particles. Environ Health Perspect.

[72] Mills NL, Miller MR, Lucking AJ, Beveridge J, Flint L, Boere AJF, Fokkens PH, Boon NA, Sandstrom T, Blomberg A (2011). Combustion-derived nanoparticulate induces the adverse vascular effects of diesel exhaust inhalation. Eur Heart J.

[73] Tao S, Xu Y, Chen M, Zhang H, Huang X, Li Z, Pan B, Peng R, Zhu Y, Kan H (2021). Exposure to different fractions of diesel exhaust PM2.5 induces different levels of pulmonary inflammation and acute phase response. Ecotoxicol Environ Saf.

[74] Vogel CF, Sciullo E, Wong P, Kuzmicky P, Kado N, Matsumura F (2005). Induction of proinflammatory cytokines and C-reactive protein in human macrophage cell line U937 exposed to air pollution particulates. Environ Health Perspect.

[75] Moller P, Scholten RH, Roursgaard M, Krais AM (2020). Inflammation, oxidative stress and genotoxicity responses to biodiesel emissions in cultured mammalian cells and animals. Crit Rev Toxicol.

[76] Weng CM, Wang CH, Lee MJ, He JR, Huang HY, Chao MW, Chung KF, Kuo HP (2018). Aryl hydrocarbon receptor activation by diesel exhaust particles mediates epithelium-derived cytokines expression in severe allergic asthma. Allergy.

[77] Podechard N, Lecureur V, Le Ferrec E, Guenon I, Sparfel L, Gilot D, Gordon JR, Lagente V, Fardel O (2008). Interleukin-8 induction by the environmental contaminant benzo(a)pyrene is aryl hydrocarbon receptor-dependent and leads to lung inflammation. Toxicol Lett.

[78] Øvrevik J, Refsnes M, Låg M, Holme JA, Schwarze PE (2015). Activation of proinflammatory responses in cells of the airway mucosa by particulate matter: oxidant- and non-oxidant-mediated triggering mechanisms. Biomolecules.

[79] Moorthy B, Chu C, Carlin DJ (2015). Polycyclic aromatic hydrocarbons: from metabolism to lung cancer. Toxicol Sci.

[80] Hiemstra PS, Grootaers G, van der Does AM, Krul CAM, Kooter IM (2018). Human lung epithelial cell cultures for analysis of inhaled toxicants: lessons learned and future directions. Toxicol In Vitro.

[81] Klein SG, Serchi T, Hoffmann L, Blömeke B, Gutleb AC (2013). An improved 3D tetraculture system mimicking the cellular organisation at the alveolar barrier to study the potential toxic effects of particles on the lung. Part Fibre Toxicol.

[82] Kletting S, Barthold S, Repnik U, Griffiths G, Loretz B, Schneider-Daum N, de Souza Carvalho-Wodarz C, Lehr CM (2018). Co-culture of human alveolar epithelial (hAELVi) and macrophage (THP-1) cell lines. Altex.

[83] Li N, Hao M, Phalen RF, Hinds WC, Nel AE (2003). Particulate air pollutants and asthma: a paradigm for the role of oxidative stress in PM-induced adverse health effects. Clin Immunol.

[84] OSHA. (n.d). Annotated TABLE Z-3 mineral dusts. Permissible exposure limits—annotated tables. https://www.osha.gov/annotated-pels/table-z-3. Retrieved 22 Nov 2021.

