# Supplementary figures and images for: The pro-inflammatory effects of combined exposure to diesel exhaust particles and mineral particles in human bronchial epithelial cells
Source: Part Fibre Toxicol. 2022 Feb 21;19:14. doi: 10.1186/s12989-022-00455-0 (PMC8862321; doi:10.1186/s12989-022-00455-0)

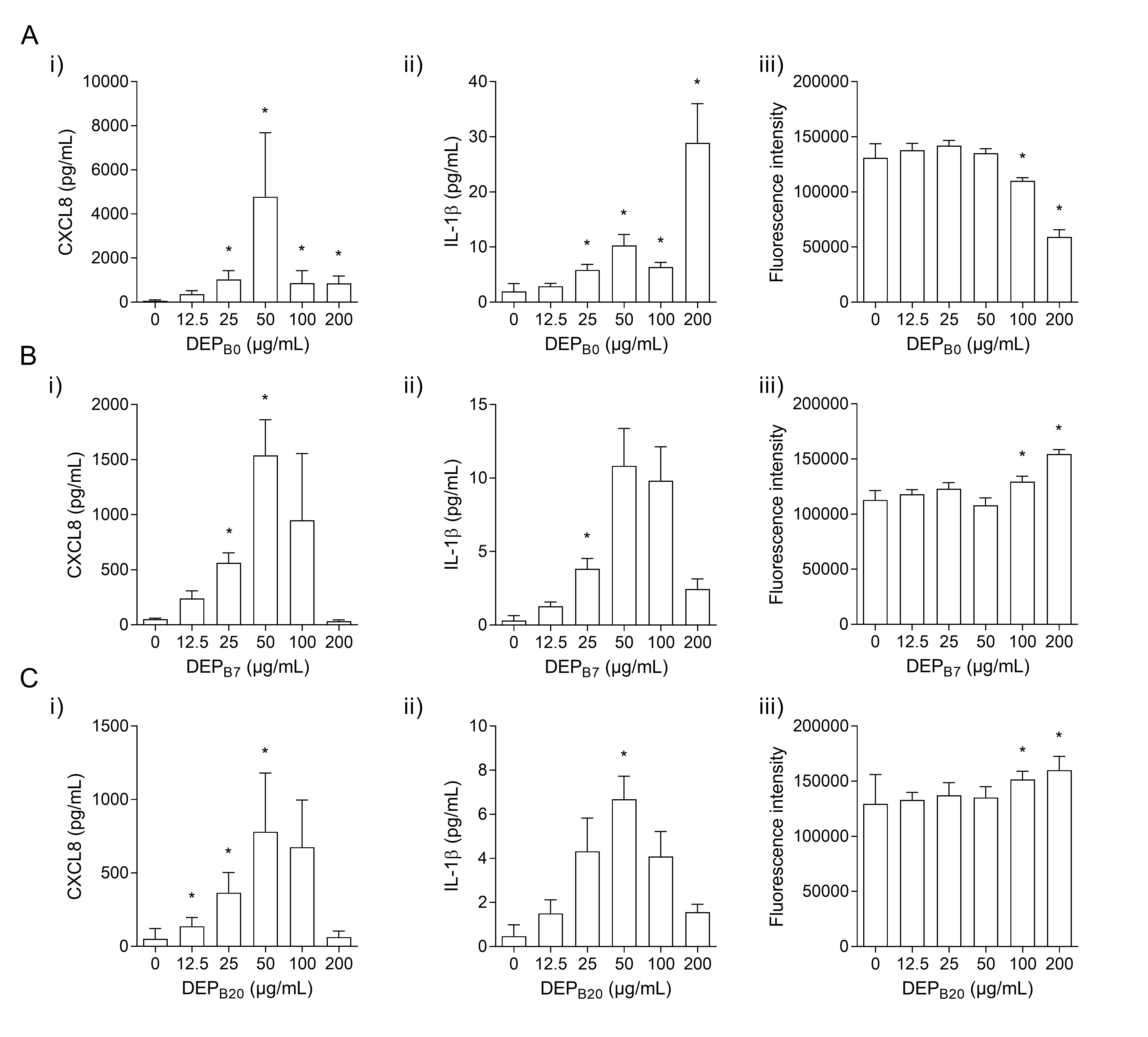

Supplement: Supplementary file 1 — Additional file 1: Figure S1. DEP-induced cytotoxicity and cytokine release. HBEC3-KT cells were exposed to 12.5–200 μg/mL (1.3–21.1 μg/cm2) DEPB0 (A), DEPB7 (B) or DEPB20 (C) for 24 h. The release of CXCL8 (i) and IL-1β (ii) in the cell culture supernatants was measured using ELISA while cell viability (iii) was determined using alamarBlue assay. Results are presented as mean ± SD (n = 3–7). Statistically significant differences were determined using one-way repeated measures ANOVA followed by Dunnett’s post-test. Values not adhering to model assumptions were log-transformed before statistical analysis. * Statistically significant difference from the respective control. [file 12989_2022_455_MOESM1_ESM.tif]

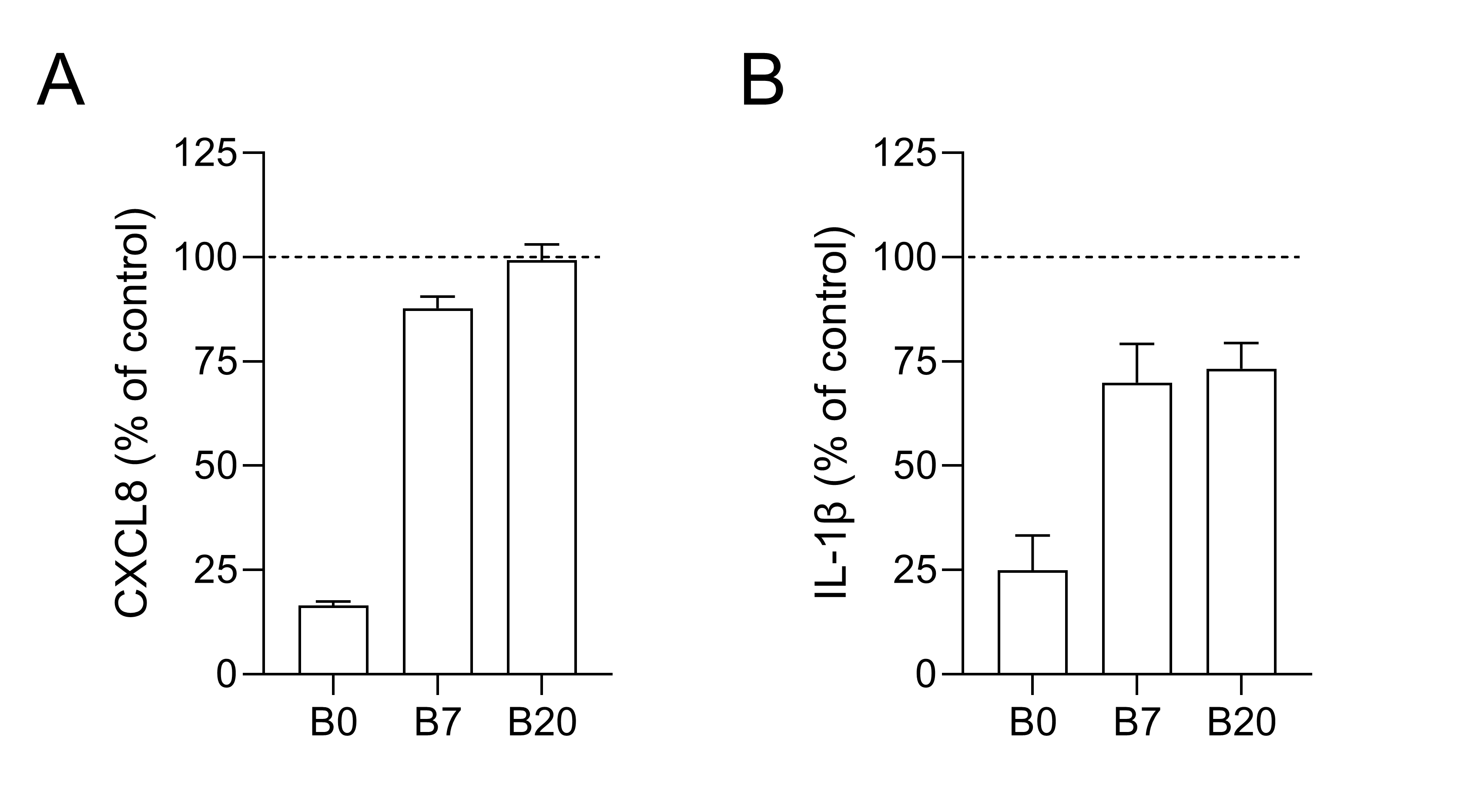

Supplement: Supplementary file 2 — Additional file 2: Figure S2. Non-specific binding of cytokines to the DEP samples. Solutions of 200 pg/mL CXCL8 (A) or 62.5 pg/mL IL-1β (B) were incubated with 50 μg/mL (5.3 μg/cm2) DEPB0, DEPB7 or DEPB20 for 24 h. The concentrations cytokines remaining in the medium were measured using ELISA. The experiment was performed in triplicate and is presented as percentage of control (mean ± SD). [file 12989_2022_455_MOESM2_ESM.tif]

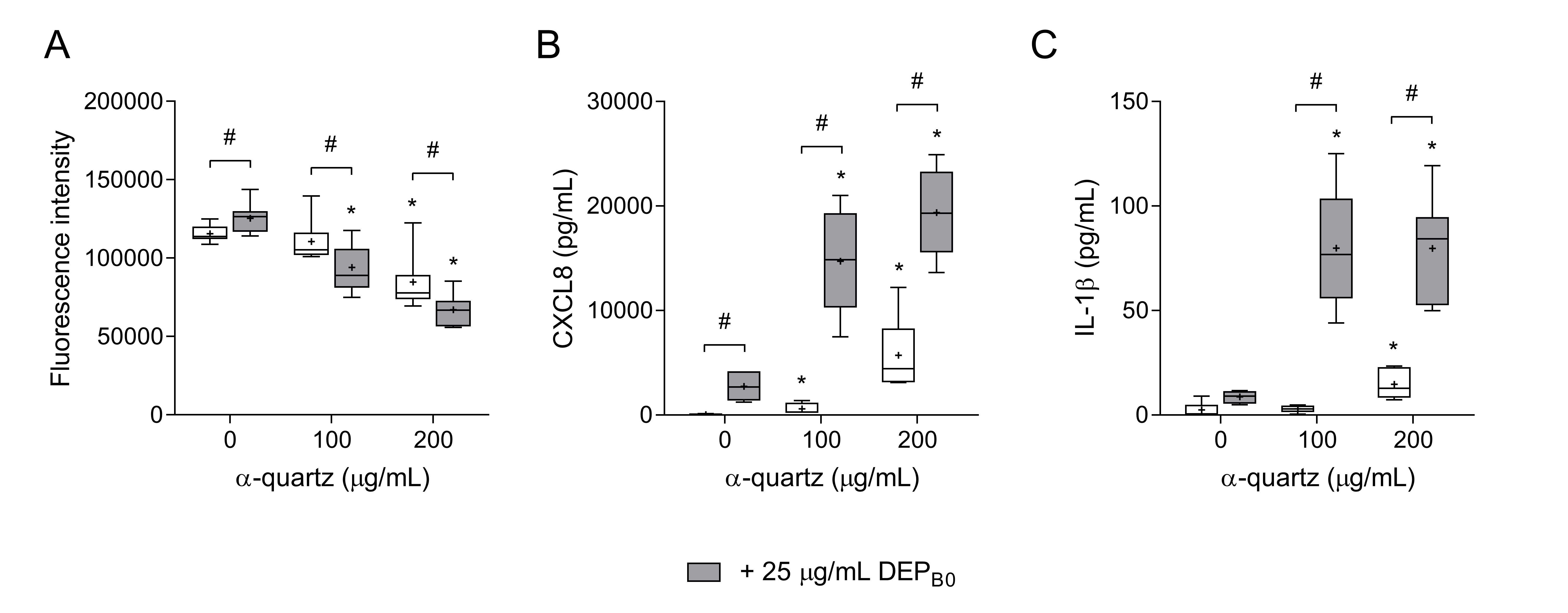

Supplement: Supplementary file 4 — Additional file 4: Figure S3. Cytotoxicity and cytokine responses after combined exposure to DEP and α-quartz at higher concentrations. HBEC3-KT cells were exposed to 25 μg/mL (2.6 μg/cm2) DEPB0 and 100–200 μg/mL (10.5–21.1 μg/cm2) α-quartz for 24 h, alone or in combination. Cytotoxicity (A) was measured by AlamarBlue assay, while the release of CXCL8 (B) and IL-1β (C) in the cell culture supernatants was measured using ELISA. Results are presented as boxplots of 6 independent experiments (Box: 25th-75th percentile, whiskers: minimum and maximum values, line: median, +: mean). Statistically significant differences were determined using two-way repeated measures ANOVA followed by Dunnett’s and Tukey post-tests. Values not adhering to model assumptions were log-transformed before statistical analysis. * Statistically significant difference from the respective control at 0 μg/mL α-quartz. # Statistically significant difference between exposure groups. [file 12989_2022_455_MOESM4_ESM.tif]

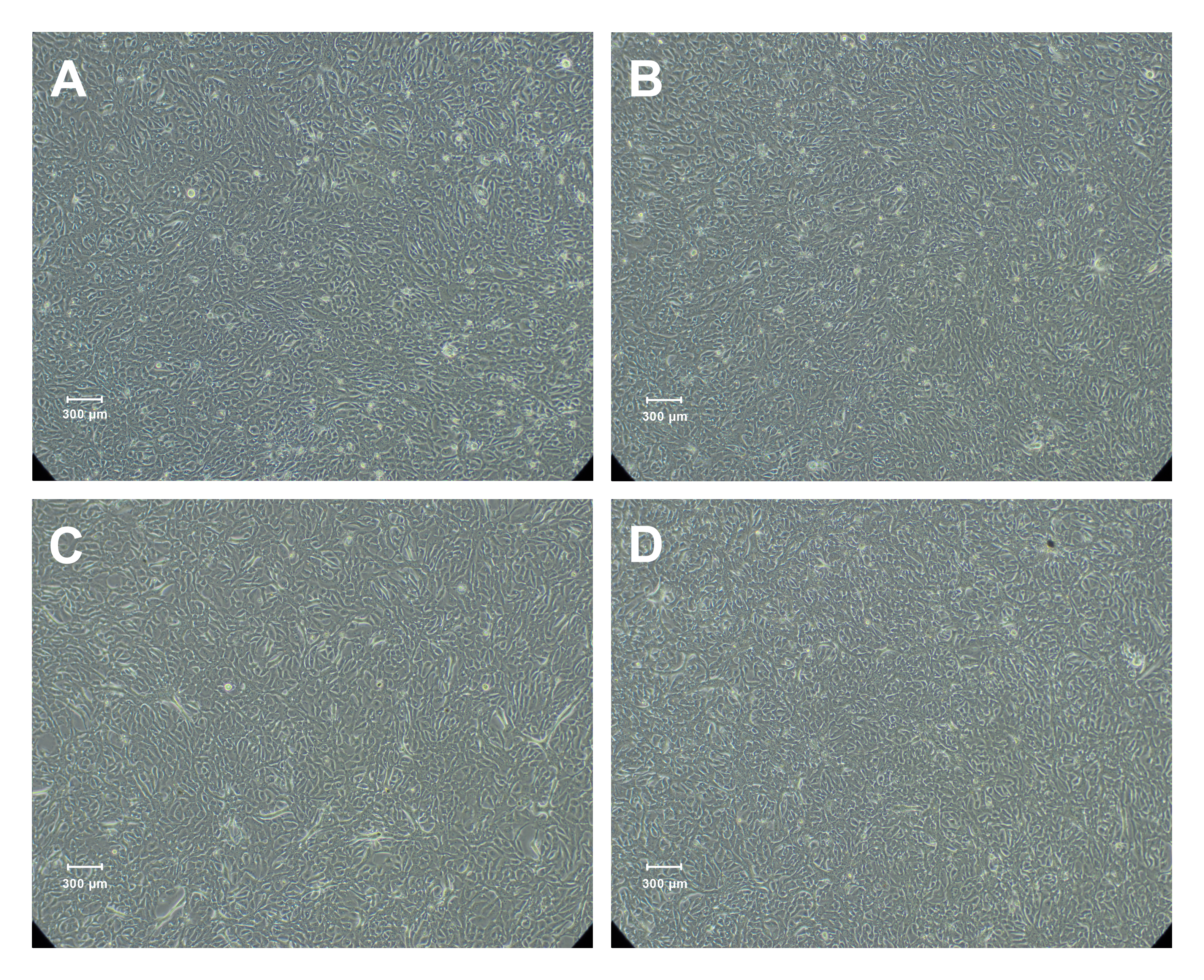

Supplement: Supplementary file 5 — Additional file 5: Figure S4. Microscope image of the cells after exposure. A microscope image of HBEC3-KT cells after exposure to vehicle (A), 100 μg/mL (10.5 μg/cm2) α-quartz (B), 50 μg/mL (5.3 μg/cm2) DEPB0 (C) or 100 μg/mL α-quartz in combination with 50 μg/mL DEPB0 (D) for 24 h [file 12989_2022_455_MOESM5_ESM.tif]
