# Supplementary material for: The pro-inflammatory effects of combined exposure to diesel exhaust particles and mineral particles in human bronchial epithelial cells
Source: Part Fibre Toxicol. 2022 Feb 21;19:14. doi: 10.1186/s12989-022-00455-0 (PMC8862321; doi:10.1186/s12989-022-00455-0)
Supplement: Supplementary file 3 — Additional file 3: Table S1. Mineralogical composition of the anorthosite, rhomb porphyry and quartz diorite samples. [file 12989_2022_455_MOESM3_ESM.docx]

Table S1. Mineralogical composition of the anorthosite, rhomb porphyry and quartz diorite samples. The content of quartz (Qtz), K-feldspar (K-feld), plagioclase (Plag), (Epi), hornblende (Horn), calcite (Calc), chlorite (Chl), and muscovite (Musc) was determined using X-ray diffraction analysis and is presented as percentages. The results are already published in Grytting et al. (2021).

|  | Qtz | K-feld | Plag | Epi | Horn | Cal | Chl | Musc |
| --- | --- | --- | --- | --- | --- | --- | --- | --- |
| Anorthosite | 0 | 0 | 61 | 13 | 0 | 0 | 3 | 23 |
| Rhomb porphyry | 4 | 33 | 46 | 0 | 7 | 2 | 6 | 2 |
| Quartz diorite | 25 | 12 | 30 | 14 | 0 | 0 | 15 | 4 |
